# When, where, and for whom do errors compound? Error sequences, contagion, and individual vulnerability in professional football

**DOI:** 10.3389/fpsyg.2026.1945783

**Published:** 2026-10-26

**Authors:** Mark R. Wilson, So Hyun Park

**Affiliations:** Department of Public Health and Sport Sciences, University of Exeter Medical School, Exeter, United Kingdom

**Keywords:** attentional control theory: sport, choking, error amnesia, error chain compounding, sequential dependence, soccer

## Abstract

Attentional Control Theory: Sport (ACTS) proposes that competitive anxiety arises from appraisals of both the cost and the probability of failure, predicting that an error should be most disruptive when the stakes are already high. We report the first large-scale test of this account in association football, drawing on 1,190,821 StatsBomb open-data event records from 330 professional matches across ten competitions. Three studies examined unforced passing errors. In Study 1 (272,381 passes, 2,202 players), situational pressure and a player’s own immediately preceding error each independently raised error probability: the odds of an error were approximately 21% higher per additional pressure component, and 57% higher following an error than following a success (OR = 1.57). The interaction, however, ran opposite to the ACTS prediction, with pressure narrowing rather than widening the post-error gap (OR = 0.91). Study 2 (54,409 post-error sequences) showed that this elevation was not confined to the player who erred: teammates taking the team’s next pass erred 3.9 percentage points above their own baseline rate, an interpersonal effect roughly half the magnitude of the within-player effect. Study 3 followed chains rather than pairs of consecutive errors. Error probability escalated monotonically with each additional consecutive prior error, from 17.7% following a success to 51.6% at a chain depth of six, and mediation analysis indicated that most of this escalation (91.7%) operated through riskier pass selection rather than degraded execution. Between-player variation in susceptibility was substantially greater for error chains than for success chains, and within every playing position the players with the lowest baseline error rates showed the steepest escalation, an association only partly accounted for by pass selection. Errors in elite football are therefore sequentially dependent, partially contagious, and unevenly damaging across players, suggesting that the cost of an error lies less in the error itself than in what it becomes if allowed to compound.

## Introduction

Elite sport performance is defined not only by the capacity for technical excellence, but by the ability to sustain that excellence when the cost of failure is highest. Errors are an inevitable part of training and competition, but their psychological impact might be weighted differently when their occurrence truly matters (Fryer et al., 2018). For many athletes, mistakes are not contained to the moment they occur; instead, their psychological impact can cascade into subsequent actions (Wilson and Eysenck, 2025). The ensuing negative spiral between anxiety and errors can lead to longer-term disruptions to both psychological well-being and performance, and potentially team crises (Jekauc et al., 2024b; Wergin et al., 2018). Understanding *why* errors might compound, *when* they are most damaging, *how far* their effects reach through consecutive sequences of actions, and *which performers* are most susceptible, are the organising questions of the present research programme.

The first research field that informs this work is that on hot and cold hand effects in sport (see Bar-Eli et al., 2006a for a review). While evidence for “hot” performance streaks remains mixed, there is stronger support for compounding error effects. For example, a consistent behavioural asymmetry emerges across three independent archival analyses of professional golf data (Elmore and Urbaczewski, 2018; Evans and Crosby, 2021; Ehrlich et al., 2025): poor performance (bogey or worse) reliably predicts a subsequent performance dip, whereas good performance (birdie or better) exerts no equivalent forward influence. Elmore and Urbaczewski (2018) observed that a prior bogey or worse increased the odds of another bogey or worse by approximately 10% on the PGA tour, and suggested that PGA Tour players have a harder time “letting go” of negative outcomes than building on positive ones. This asymmetry is theoretically important as it suggests that the psychological impact of failure is not the mirror image of success but carries a distinct and heavier cognitive cost (Baumeister et al., 2001). There is an evolutionary advantage for this “negativity bias” (Vaish et al., 2008), as avoiding harm carries a greater survival premium than pursuing an equivalent reward (Norris, 2021). However, the same bias also shapes how people appraise themselves and their prospects and is implicated in more negative self-evaluation and heightened threat perception (Müller-Pinzler et al., 2019).

Crucially, this bias may paradoxically penalise the most accomplished competitors. Evans and Crosby (2021) observed that more successful professional golfers (split at the median for scoring ability) were *more* susceptible to these performance dips after recent failure. The cold-hand coefficients for prior bogey and prior double-bogey-or-worse were statistically significant for the better-than-median players, but not for the worse-than-median players (Evans and Crosby, 2021). Because errors are rarer for elite performers, a mistake may represent a more profound violation of expectations (Hajcak, 2012) than one committed by a less-skilled performer for whom failure is more schema-congruent. More accurate performers hold competence-related self-views with greater certainty and accumulated evidential support (Swann, 2012) and stronger associated self-guides (“I shouldn’t make mistakes”; Higgins, 1987). The psychological cost of threats to a self-concept heavily anchored in technical reliability, potentially heightens any negativity bias for errors. Furthermore, while a single error may be ambiguous enough to attribute to chance, each further consecutive error is likely to shift error attributions from unstable and external toward being more stable and internalised (Weiner, 1985). Therefore, each additional error in a chain should compound the disruption caused by the last. Taken together, vulnerability to error compounding may be influenced by a performer’s own standard of expected competence.

A second literature base has also analysed large scale archival data to document the performance costs of situational pressure (choking under pressure; Mesagno et al., 2021). Drawing on the logic that the stakes attached to any given action can be objectively quantified from match context, researchers have consistently shown that athletes underperform when the cost of failure is highest. Hickman and Metz (2015) analysed individual putts on the final hole of PGA Tour tournaments and found that as the amount of money riding on a shot increased, the likelihood of making it was significantly reduced. Toma (2017) demonstrated comparable choking effects across over two million free-throw attempts in college and professional basketball, finding that players were less likely to convert in the final 30 s of close games. Bucciol and Castagnetti (2020) showed that archery scores were lower in tiebreak situations, and especially if the tiebreak took place in a prestigious world tournament.

These parallel streams of evidence establish that pressure and errors impair performance but cannot determine any potential multiplicative effects; a core tenet of Attentional Control Theory: Sport (ACTS; Eysenck and Wilson, 2016; Wilson and Eysenck, 2025). In line with its parent theory, Attentional Control Theory (ACT; Eysenck et al., 2007; Eysenck et al., 2023), ACTS states that anxiety disrupts the balance between goal-directed and stimulus-driven attentional systems, increasing distractibility and reducing processing efficiency. ACTS further proposes that competitive anxiety emerges not from situational pressure alone, but from the performer’s appraisal of two interacting factors (Berenbaum, 2010): the cost of failure (what is at stake?) and the *probability* of failure (how am I doing?). These appraisals are not static and update dynamically in response to ongoing performance. An error during performance may raise the perceived probability of future failure and when this occurs in a context where the cost of failure is also elevated, the interaction should increase the psychological disruption (Wilson and Eysenck, 2025).

Harris and colleagues provided the first test of this proposed interaction using archival data in American football (2019) and professional tennis (2021). Across more than 212,000 NFL plays and 650,000 Grand Slam tennis points, both studies demonstrated that (a) pressure increased the probability of errors, (b) prior errors predicted subsequent errors, and (c) the compounding impact of a prior error was significantly increased under conditions of elevated pressure. In tennis, the probability of an unforced error doubled when players had erred on their previous shot, *and* the point was high-pressure (Harris et al., 2021). While a recent study by Klingner et al. (2023) testing the predictions of ACTS in elite surfing, only found support for the main effect of a prior error on subsequent ride performance (not for pressure or the interaction), further archival research testing these predictions in different sports is warranted.

A limitation of the existing evidence base examining performance dependencies is that they have been conducted in sports with highly structured, discrete sequences. Association football is an interdependent, continuous team sport where performance is collective, and responsibilities are distributed across players in real time. If, as ACTS proposes, an error updates a performer’s own appraisal of failure probability, a similar process may extend beyond the individual who erred. Neurocognitive research examining ‘error observation’ points to shared neural machinery for self- and other-error detection (Musco et al., 2023). Furthermore, experimental dyad studies have revealed that a perceived decline in a partner’s competence can impair the observer’s own performance (Boss and Kleinert, 2015). A teammate’s visible error could therefore act as a social signal that the immediate context has become more threatening, or that collective performance is deteriorating (Wergin et al., 2025). Wergin et al.’s (2018) qualitative examination of collective sport team collapses points to attentional focus and pressure as key antecedents that lead to unforced error accumulation and subsequent decreased performance contagion. These mechanisms therefore align with those of ACTS and support a bi-directional relationship between pressure, errors, and psychological disruption.

Three interrelated studies sought to extend our understanding of the impact of compounded errors on sport performance by applying the analytical logic of ACTS to professional football. Study 1 tested the foundational ACTS prediction that errors and pressure exert a multiplicative influence on subsequent performance, replicating the approach of Harris et al. (2019, 2021) in a new sporting context. The aim was to determine whether football, as a continuous and team-based game, produces the same error-compounding signature documented previously. Study 2 took advantage of the interdependent nature of team performance in football to examine a contagion hypothesis for a team sport. Do errors spread interpersonally between teammates (the “where” of error compounding; Wergin et al., 2018)? Study 3 moved beyond examining immediately preceding task performance to examine the effect of accumulated failure history. Does the probability of error escalate as players move deeper into unbroken chains of failure, and do players differ in their vulnerability to this escalation? Together, the studies move from detecting error compounding at the individual level, to tracing its spread across teammates, to characterising its depth and individual variation, extending ACTS-based archival research into the world’s most widely played sport for the first time.

### Study 1: Replication. Do errors beget errors in football?

Study 1 tested the three core predictions of ACTS (Eysenck and Wilson, 2016; Wilson and Eysenck, 2025): (1) that situational pressure increases the probability of an unforced error, (2) that prior errors elevate the probability of a subsequent error within the same player, and (3) that these two effects combine multiplicatively such that the prior-error effect is increased under elevated pressure. The design followed the analytical logic of Harris et al. (2019, 2021) applied to StatsBomb open event data for a sample of professional football matches.

#### Method

Association football presents additional methodological challenges compared to the sports studied previously. Its continuous, interdependent gameplay does not have the natural discrete boundaries associated with NFL plays and tennis points when assessing dependence. We focused on passing as the most frequent and distributed offensive action with ~1,000 incidents per match in our sample. Unforced pass actions (removing passes that were physically pressed by an opponent, see Measures) were selected as the closest football equivalent to the unforced errors examined in tennis (Harris et al., 2021) and the controllable play failures examined in American football (Harris et al., 2019).

A further complication is that both pressure and errors might influence the decision of which pass to attempt (selection) as well as the execution of that pass once chosen (pass success). Indeed, in one of the few field studies of passing performance under pressure, Jackson et al. (2013, Study 2) found that decision-specific reinvestment predicted the drop in netball passing accuracy from low- to high-pressure matches but acknowledged their outcome measure could not distinguish poorer decision-making from degraded execution. To begin to untangle what was attempted from how well it was executed, we drew on pass difficulty models that estimate a pass’s intrinsic success probability (e.g., Anzer and Bauer, 2022) and included this as a covariate in exploratory analyses.

Finally, playing position was included as a supplementary covariate as it influences pass volume (Sarmento et al., 2024) and may shape the task constraints under which passes occur (Araújo et al., 2006). We therefore treated position^1^ as a fixed-effect control and additionally tested position-by-prior-error interactions in supplementary analyses to establish whether compounding generalised across roles.

All analysis code (Python and R) for the three studies is available on OSF to support reproducibility.^2^ Generative AI assistance in the development of this code is disclosed in the Generative AI statement.

##### Data source

Event-level match data were obtained from StatsBomb’s open data repository (StatsBomb, 2024), which provides detailed, manually coded records of every on-ball action in professional football matches. Each event record contains the action type (e.g., pass, shot, dribble), outcome, location coordinates, timestamp, player identity, and contextual variables including possession sequence membership and a binary indicator of whether the player was under immediate physical pressure from an opponent at the moment of the action.

The dataset comprised all available matches from 10 competitions across the StatsBomb open data catalogue: La Liga (2017/18 through 2020/21; four seasons), the Premier League (2003/04), the UEFA Champions League (2017/18 and 2018/19), the 2018 FIFA World Cup, the 2019 FIFA Women’s World Cup, and the 2018 National Women’s Soccer League (NWSL). In total, the sample contained 330 matches (113 unique teams and 2,252 unique players) yielding 1,190,821 individual events^3^. The multi-competition design provided heterogeneity in playing context (domestic league, international tournament, knockout vs. group stage, men’s and women’s football) while maintaining consistency in data coding methodology.

#### Measures

##### Unforced pass actions

333,918 passes could be assigned a success/error label (via our is_classifiable indicator), which excluded 263 events (0.08%) coded by StatsBomb as “Injury_Clearance”. Passes were further restricted to those made without immediate physical pressure from an opponent, based on StatsBomb’s manual under_pressure coding. The exclusion of ‘forced’ errors was designed to isolate unforced errors from passes that failed because of defensive intervention, and excluded approximately 15.7% of classifiable passes, yielding 281,560 unforced pass actions for the primary analyses.

##### Outcome classification (error/success)

An unforced pass was classified as an error if it did not result in a successful completion to a teammate; if StatsBomb’s pass_outcome field was coded as (1) incomplete (16.6% of passes), (2) out (1.8% of passes), (3) offside (0.4% of passes), or (4) unknown (0.6% of passes). Completed passes were classified as successes (80.5% of passes), reflecting an error rate of 19.5%. For each unforced pass action, the outcome of the player’s own immediately preceding unforced pass within the same match was coded as a binary prior error indicator. The comparison was therefore specifically between a prior unforced pass error and an unforced pass success, not across all actions. Each player’s first unforced pass in each match was excluded for lack of a valid prior, yielding a primary dataset of 272,381 unforced pass actions with valid prior-action data.

##### Four-component pressure index

Situational pressure was operationalised using a four-component additive index, designed to capture cost-of-failure contexts in which the consequences of an error are elevated. The four components were summed to produce a single composite ranging from 0 to 4, capturing the number of co-occurring cost-of-failure markers active at the moment of each action. Equal additive weighting was chosen for parsimony and to mirror the pressure-scoring approach of Harris et al. (2019, 2021). The four components and their prevalence were: (1) “Trailing” on the scoreboard at the moment of the action (25.7%); (2) “Closing-in-balance”, operationalised as the joint condition of ≥ 75 min elapsed (StatsBomb’s continuous match-clock minute field, which was not reset at the start of extra-time periods)^4^ and a goal difference of ≤ 1 goal (11.6%); (3) ‘Knockout match’, indicating that the match was a tournament knockout fixture rather than a group-stage or league fixture (9.9%); and (4) ‘Numerical disadvantage’, indicating that the player’s team was playing with fewer outfield players than the opposition following a red card to a teammate (1.18%; concentrated in 38 of 330 matches). Running match scores were reconstructed from exact goal timestamps in the StatsBomb data; knockout match status was determined from the competition_stage metadata; and numerical disadvantage was computed from cumulative red-card events for each team prior to each action.

Each component was selected to index a distinct, previously-documented source of elevated stakes rather than a single underlying construct measured four times. We selected contexts where unexpected performance errors have been previously related to meaningful failure (e.g., Fryer et al., 2018). Trailing and closing-in-balance both reflect scoreline-based stakes shown to depress performance in comparable late-game and close-game contexts in football (e.g., Indaco and Teqja, 2025; Santos, 2024). Knockout match status indexes acute, non-recoverable stakes with precedent in elimination contexts where reduced win probabilities have been shown (e.g., in archery, Bucciol and Castagnetti, 2020 and basketball, Morgulev and Galily, 2018). Numerical disadvantage effects from red cards are well established in football (e.g., Červený et al., 2018) and have been related explicitly to psychological performance crises (Bar-Eli et al., 2006b). We tested whether this composite masks four separate constructs by entering each component individually in Study 2 (Model 3), and confirm the composite-level estimates are not driven by any single marker through a series of specification checks in Supplementary Table S1 (isolating trailing from the remaining three components; adding a defensive-third covariate; removing position fixed effects; and re-estimating the index with numerical disadvantage excluded entirely).

##### Pass-difficulty covariates

Three pass-difficulty covariates were also extracted for post-hoc mediation analyses to untangle potential changes in what was attempted from how well passes were executed. (1) Pass length was the Euclidean distance in StatsBomb pitch units between the action location and the pass end location, with a log transformation applied to reduce right-skew. (2) The forward-pass indicator was a binary flag for whether the pass moved the ball forward (pass_end_x > action_x, expressed left-to-right from the acting team’s perspective). (3) Pass height was a three-level categorical variable (ground, low, high) as coded by StatsBomb, with ground passes as the reference category.

#### Analytical strategy

Two logistic regression models were estimated on the binary unforced-pass error outcome. Because events are nested within players and within matches, standard errors were two-way cluster-robust, clustered simultaneously on player and on match (Cameron et al., 2011)^5^. The primary model (Model 1) regressed the probability of an unforced pass error on the four-component pressure index, the prior-error indicator, and their interaction. The supplementary mediation model (Model 2) added the three pass-difficulty covariates to the Model 1 specification. Comparison of the coefficients across the two models allowed us to test how much of the pressure and prior-error effects operate through the difficulty of the pass being attempted versus through pure execution failure on otherwise comparable passes. Odds ratios (OR) with 95% confidence intervals are reported as effect sizes throughout. A supplementary sensitivity analysis (Supplementary Table S1) examined the robustness of the pressure × prior-error interaction estimate across alternative reasonable model specifications. All analyses were conducted in Python using statsmodels (Seabold and Perktold, 2010).

#### Results

##### Data overview

The primary dataset comprised 272,381 unforced pass actions with valid prior-action data from 330 matches and 2,202 unique players. The overall error rate for this sample was 19.4%. The pressure index had a mean of 0.48 (SD = 0.66), with 60.2% of actions occurring at P = 0 (no pressure markers active), 32.0% at P = 1, 7.1% at P = 2, 0.6% at P = 3, and 0.01% at P = 4. Marginal error rates rose monotonically with the pressure index, from 17.8% at P = 0 to 24.2% at P = 3.

##### Primary replication (model 1)

The primary logistic regression revealed significant main effects in the directions predicted by ACTS. Pressure significantly increased the probability of an unforced pass error (OR = 1.21, 95% CI [1.15, 1.27], *p* < 0.001) with each additional pressure component raising the odds of an unforced error by approximately 21%. A prior error on the player’s own immediately preceding unforced pass also significantly predicted a subsequent error (OR = 1.57, 95% CI [1.46, 1.68], *p* < 0.001); the odds were approximately 57% higher following an unforced pass error than a success. In contrast to Harris et al. (2019, 2021), however, the pressure × prior-error interaction narrowed the post-error gap, rather than widening it as ACTS predicts (OR = 0.91, 95% CI [0.87, 0.95], *p* < 0.001). Each additional pressure component reduced the multiplicative prior-error effect by approximately 9%, although the pattern was not strictly monotonic across the four pressure levels (Spearman r = −0.80). With only four ordered points this trend is descriptive rather than a basis for formal inference. The P = 3 estimate is also based on a comparatively small subsample (*n* = 1,767; see Figure 1 and Supplementary Table S2 for all values).

**Figure 1:**
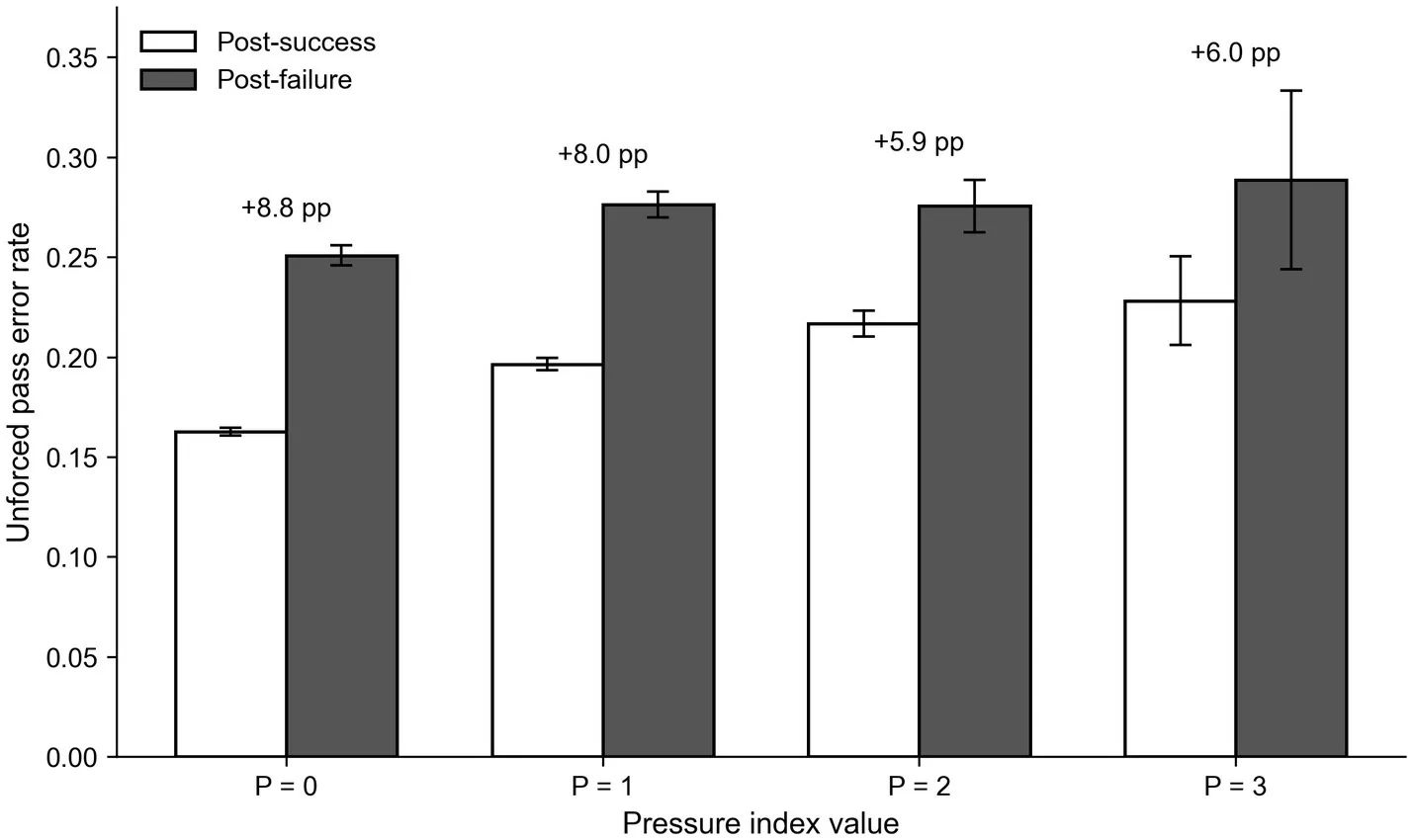
Unforced pass error rate as a function of pressure index value and prior-action outcome. Bars show mean error rate within each pressure level for actions following a successful previous action (white bars) and actions following an unforced pass error by the same player in the same match (dark bars). Error bars represent 95% confidence intervals. Values above bar pairs indicate the post-error vs. post-success gap in percentage points. *N* = 272,381 unforced pass actions.

The prior-error effect was evident within every position, ranging from *OR* = 1.18 in strikers to *OR* = 2.23 in goalkeepers (all *p* < 0.01), indicating that compounding is not an artefact of positional composition (Supplementary Figure S1; Supplementary Table S3b).

##### Pass selection mediation (model 2)

Pass difficulty exerted very large effects on the probability of an unforced error: (1) high passes had approximately 15 times the odds of failing of ground passes (OR = 15.01, 95% CI [13.86, 16.26], *p* < 0.001); (2) low passes approximately 4 times more error-prone (OR = 3.98, 95% CI [3.73, 4.25], *p* < 0.001); and (3) forward passes were 3.4 times more error-prone than non-forward passes (OR = 3.40, 95% CI [3.26, 3.55], *p* < 0.001). Adjusting for pass difficulty reduced but did not eliminate the pressure and prior-error effects from Model 1 (see Figure 2 for odds ratio changes).

**Figure 2:**
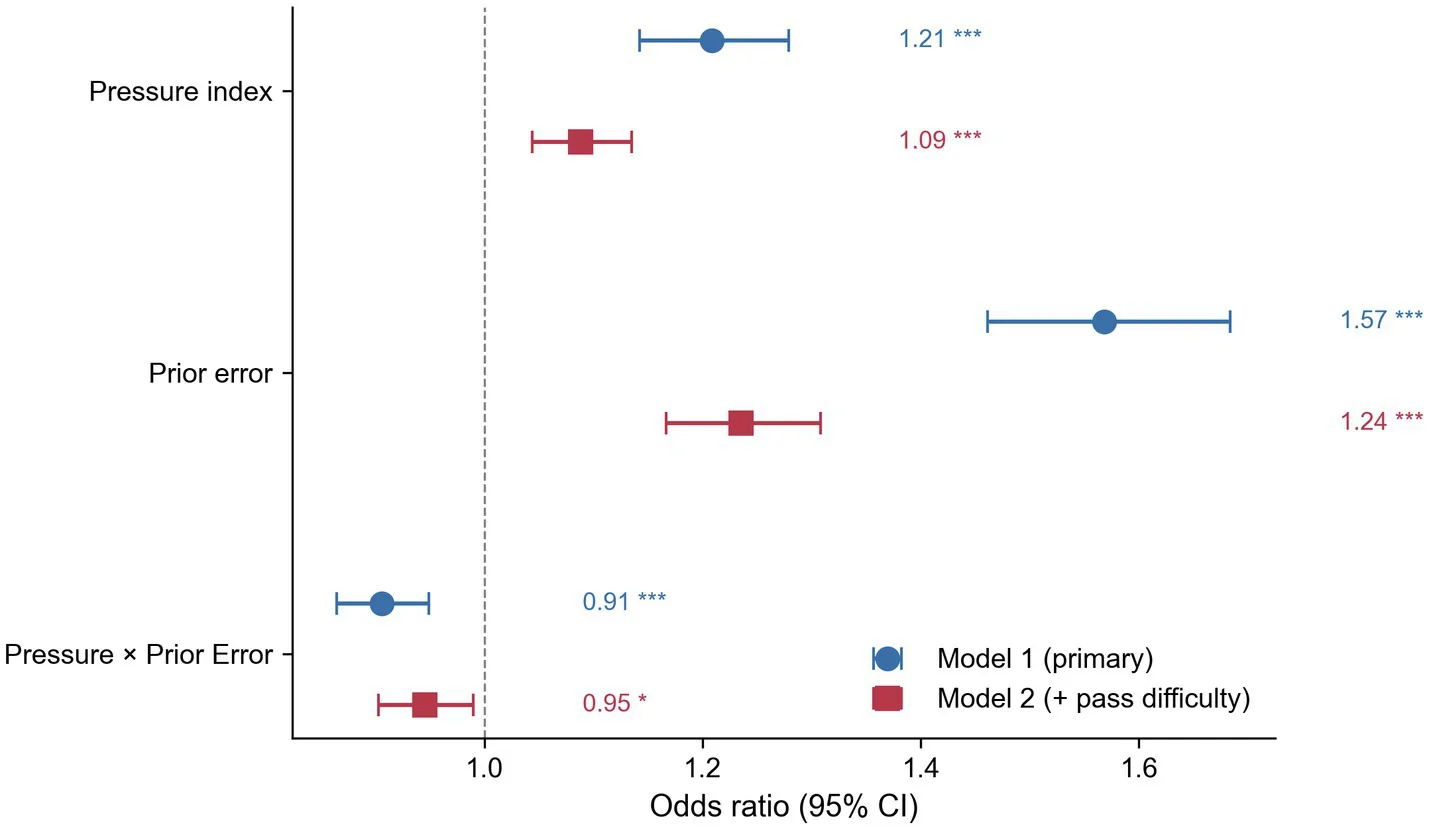
Coefficient comparison between the primary model (Model 1) and the supplementary pass-difficulty model (Model 2) for the three core predictors. Points are odds-ratio estimates; horizontal bars are 95% confidence intervals; the vertical dashed line marks OR = 1 (no effect). All three coefficients are reduced in magnitude after pass-difficulty adjustment but remain statistically significant, indicating that pass selection mediates a substantial share of each effect without eliminating any of them. *** *p* < 0.001, * *p* < 0.05.

Using the Karlson-Holm-Breen (KHB) decomposition, which corrects for the rescaling artefact that affects naive coefficient comparisons across nested logistic models (Karlson et al., 2012), pass-difficulty covariates mediated 59.0% of the pressure main effect, 58.8% of the prior-error main effect, and 43.1% of the interaction’s narrowing. Full coefficient estimates for the pass-difficulty model are reported in Supplementary Table S3.

#### Discussion

The study is the first to test the predictions of ACTS (Eysenck and Wilson, 2016) in large-scale football data, replicating an approach previously adopted by Harris and colleagues (Harris et al., 2019, 2021). Players were more likely to commit an unforced pass error when situational pressure was elevated, and immediately following an unforced error, replicating the basic pressure and compounding effects observed in NFL (Harris et al., 2019) and elite tennis (Harris et al., 2021). However, pressure narrowed rather than widened the prior-error effect, with the post-error vs. post-success gap smaller at higher pressure index values (P3/P4) than lower values (P1/P2). We treat the magnitude of this narrowing cautiously. The interaction was robust to the inclusion of an additional spatial covariate (defensive-third location^6^) and to removing positional fixed effects (Supplementary Table S1, Specifications C and E). However, separating trailing from the composite index reduced the interaction to non-significance (Supplementary Table S1, Specifications B and D; OR = 0.96, *ps* = 0.183 and 0.181), indicating that trailing drives most of this narrowing rather than the pressure index uniformly.

One plausible explanation of this departure from the NFL and tennis findings is that the four-component pressure index is not an adequate proxy for the moment-to-moment psychological pressure that ACTS theorises. In NFL and tennis, the down-and-distance/point-by-point structure makes each play’s stakes explicit and discrete, whereas the components may not capture the rapid fluctuation of perceived stakes that occur within football’s passages of play. Future research may want to explore how continuous measures that update within possession sequences could better index “pressure” in football (e.g., expected possession value EPV; Fernández et al., 2019 and valuing actions by estimating probabilities VAEP; Decroos et al., 2019). While we accounted for the effect of momentary *physical* pressure on the passer (via StatsBomb under_pressure coding) other contextual influences may bear on this interaction (e.g., how the opposition press after an error). We did not add such controls. Post-error context is partly a consequence of the error itself, so adjusting for the press would remove part of the effect under study. The open StatsBomb data do not in any case carry tactical detail at that resolution.

The pass difficulty mediation analyses provide an important nuance for our headline findings and therefore extend previous research. Players under elevated pressure, and immediately following an error, were attempting harder passes than in lower-pressure or post-success contexts, and this pass selection shift accounted for ~60% of both main effects. This pattern may be due to impaired decision quality (Jackson et al., 2013) but is also consistent with a shift in risk preference. Players may become more willing to attempt higher-value, harder passes under pressure and after an error. A parallel shift is evident at team level: across the five major European leagues, trailing teams intensify their attacking play while leading teams grow more conservative (Yang and Zhao, 2023). The tendency for a recent negative outcome to increase risk-seeking also has a long precedent in behavioural economics. A person who has experienced a recent loss or error is more likely to accept a high-risk gamble if it offers a specific path to erase that deficit; a phenomenon Thaler and Johnson (1990) named the ‘break-even effect’. In contrast to its mediating role on the pressure and error main effects, pass selection explained less than half of the interaction effect narrowing, leaving execution as the larger contributor. Taken together, the three effects are not a single phenomenon with one mechanism but a combination of pass-selection and execution influences whose relative weight shifts across effects. What our observational design cannot establish is why the pass-selection share arises; whether harder passes reflect degraded decision quality or a deliberate shift in risk preference (Jackson et al., 2013).

Even though pass-difficulty adjustment reduces the prior-error effect from OR = 1.57 to 1.24, it remains larger than the NFL (OR = 1.09; Harris et al., 2019) and tennis (OR = 1.20; Harris et al., 2021) estimates, neither of which adjusted for play or shot difficulty. The findings of Study 1 therefore provide cause to focus subsequent analyses more directly on the error signal itself rather than on the cost-of-failure pressure context within which it occurs.

### Study 2: Error contagion. Are errors infectious?

#### Overview

Study 1 established that at the individual level, a prior error significantly increases the probability of the next action also being an error. Athletes themselves describe an analogous process at the team level, where one player’s mistakes “infect” teammates’ performance (Wergin et al., 2018). The appraisal mechanism proposed by ACTS offers a theoretical account of how this might occur. Study 2 tested this directly: Does one player’s error elevate the probability that the next teammate to touch the ball also errs? Because performance accomplishments are a stronger source of self-efficacy than vicarious experience (Bandura, 1982), any interpersonal effect would be expected to be smaller in magnitude than the within-player prior-error effect.

#### Method

##### Sample

Study 2 drew on the same underlying StatsBomb corpus described in Study 1 (330 matches across the 10 competitions, including 1,190,821 total events). The Study 2 analytic sample comprised 281,560 unforced pass actions (classifiable passes with no defender pressure flagged) by 2,233 unique players. There were 19 players among the full dataset of 2,252 who did not make an unforced classifiable pass. A larger sample than Study 1’s 2,202 players was used, because Study 2 does not require a valid prior action and retains each player’s first unforced pass per match (unlike Study 1’s lag-1 model).

#### Variables

##### Follow-up passes

For each unforced pass error (the source error), we identified the next unforced pass by any player on the same team in the same match (the follow-up pass), yielding 54,409 source-error → follow-up pairs. In most cases a teammate took the follow-up pass (different-player pairs; *n* = 47,288, 86.9%). In the remainder, the player who made the source error took it themselves (same-player pairs; *n* = 7,121, 13.1%). Follow-up passes were almost exclusively (99.8%) post-turnover, capturing a team’s first action after regaining the ball following at least one intervening event by the opposition. The outcome measure was the binary error status of the follow-up pass.

#### Analytical strategy

The primary predictor was whether the follow-up pass was taken by the source player themselves (same-player) or by a teammate (different-player). Following the specifications used in Study 1, the four-component pressure index (measured at the follow-up action), the three pass-difficulty covariates and the seven positional fixed effects, served as controls and mediators.

Four logistic regressions were fitted. Model 1 estimated the same-player effect with the pressure index and positional controls. Model 2 added the same-player × pressure interaction. Model 3 replaced the composite pressure index with its four components and added the four corresponding same-player × component interactions. Model 4 added the three pass-difficulty covariates to the Model 1 specification. As in Study 1, all models used two-way cluster-robust standard errors, clustered on the player taking the follow-up pass and on match (Cameron et al., 2011). Unadjusted differences in error rate between same-player and different-player follow-ups were tested by a Pearson chi-square test of independence.

A one-sample t-test isolated the elevation observed in *different*-player follow-ups from individual differences in which players tend to receive them. Each player’s baseline error rate was computed across all their unforced passes in the full dataset and the residual for each different-player follow-up was defined as the difference between the observed error rate and their own baseline. A one-sample *t*-test against zero assessed whether the mean residual was reliably positive.

#### Results

Error rates following a source error were elevated relative to the unconditional baseline of 0.19 in both follow-up conditions (Figure 3). The error rate when the source player took the follow-up pass themselves (*n* = 7,121) was 0.287, an absolute elevation of +9.3 percentage points (pp). The error rate when a teammate took the follow-up pass (*n* = 47,288) was 0.272, an elevation of +7.8 pp. The same-versus-different rate gap of approximately +1.5 pp. was small but statistically reliable in the unadjusted comparison (χ^2^(1) = 6.72, *p* = 0.010), indicating a modest additional elevation when the source player rather than a teammate took the next action. The residual contagion test established that the elevation observed in different player follow-ups was not simply attributable to individual differences in who takes the ball after a teammate’s error (mean residual = +3.93 pp (*SE* = 0.20 pp), *t*(47,287) = 19.71, *p* < 0.001).

**Figure 3:**
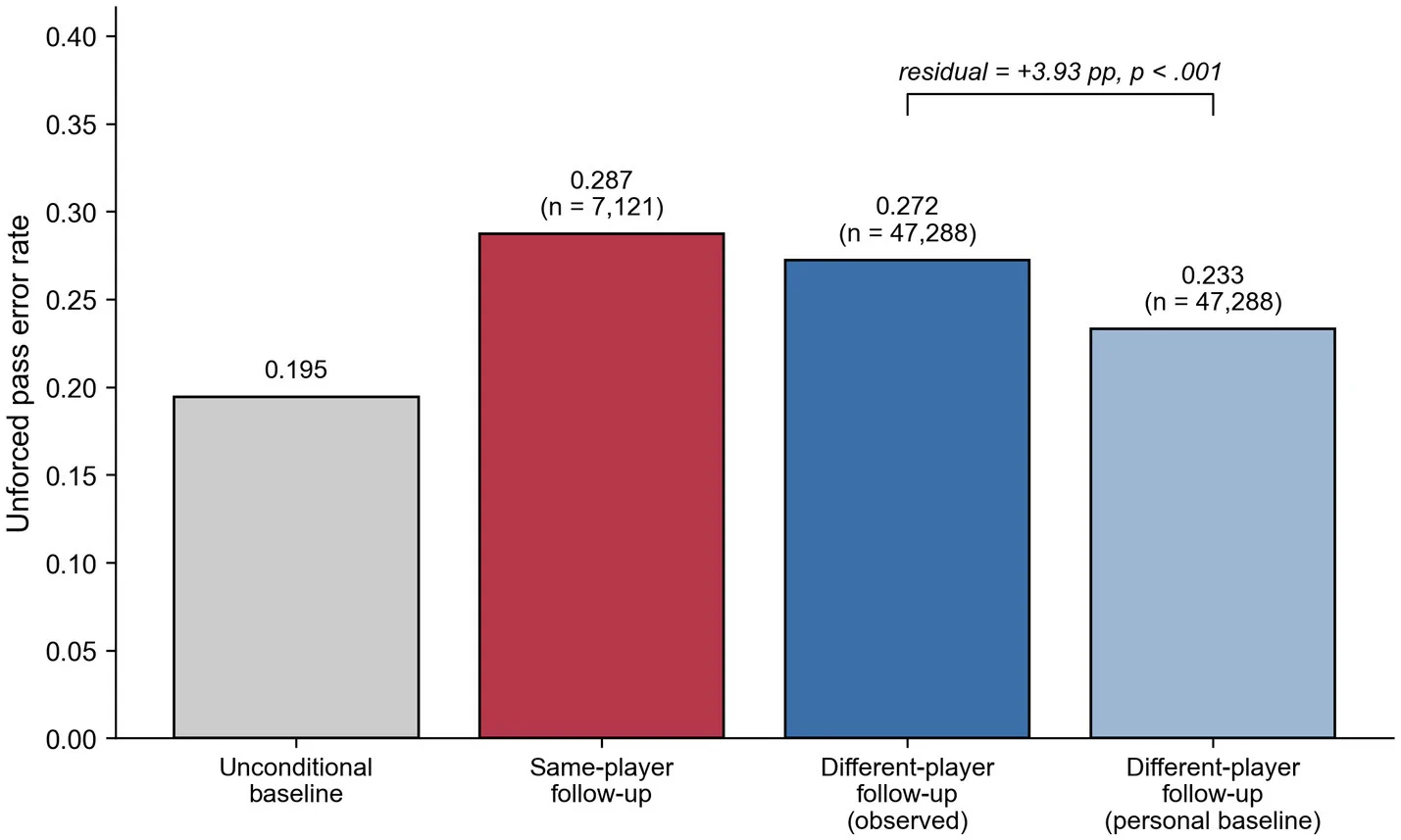
Unforced pass error rates across follow-up conditions. Bars show the unconditional baseline computed across all unforced passes, the error rate in same-player follow-ups, the error rate in different-player follow-ups (observed), and the mean of those players’ own baseline error rates computed across all their unforced passes in the dataset. The bracket above the third and fourth bars indicates the residual contagion: the mean within-player difference between observed and baseline error rates in different-player follow-up passes.

The adjusted same-player coefficient was small across all specifications (Figure 4). In Model 1 it was *OR* = 1.11, 95% CI [1.04, 1.19], *p* = 0.003, with the pressure index also raising error probability (*OR* = 1.09, 95% CI [1.03, 1.15]). Pressure did not moderate the same-player effect (Model 2: same-player × pressure interaction *OR* = 0.99, 95% CI [0.91, 1.09], *p* = 0.855). Decomposing the pressure index into its four components (Model 3) similarly returned no significant moderation of the same-player effect by any single component (all four interactions *ps* ≥ 0.123). When the three pass-difficulty covariates were added (Model 4), the same-player coefficient was reduced to *OR* = 1.03, 95% CI [0.96, 1.11], *p* = 0.380, or fully absorbed by what was attempted on the next pass. The pass difficulty covariates carried large coefficients: high passes had 9.0 times the odds of failing of ground passes (OR = 9.03, 95% CI [8.20, 9.94]), low passes 2.4 times the odds, and forward passes 3.2 times the odds of non-forward passes (95% CI [3.0, 3.5]). Longer passes carried a small multiplicative penalty (OR = 1.23 per log-unit of pass length).

**Figure 4:**
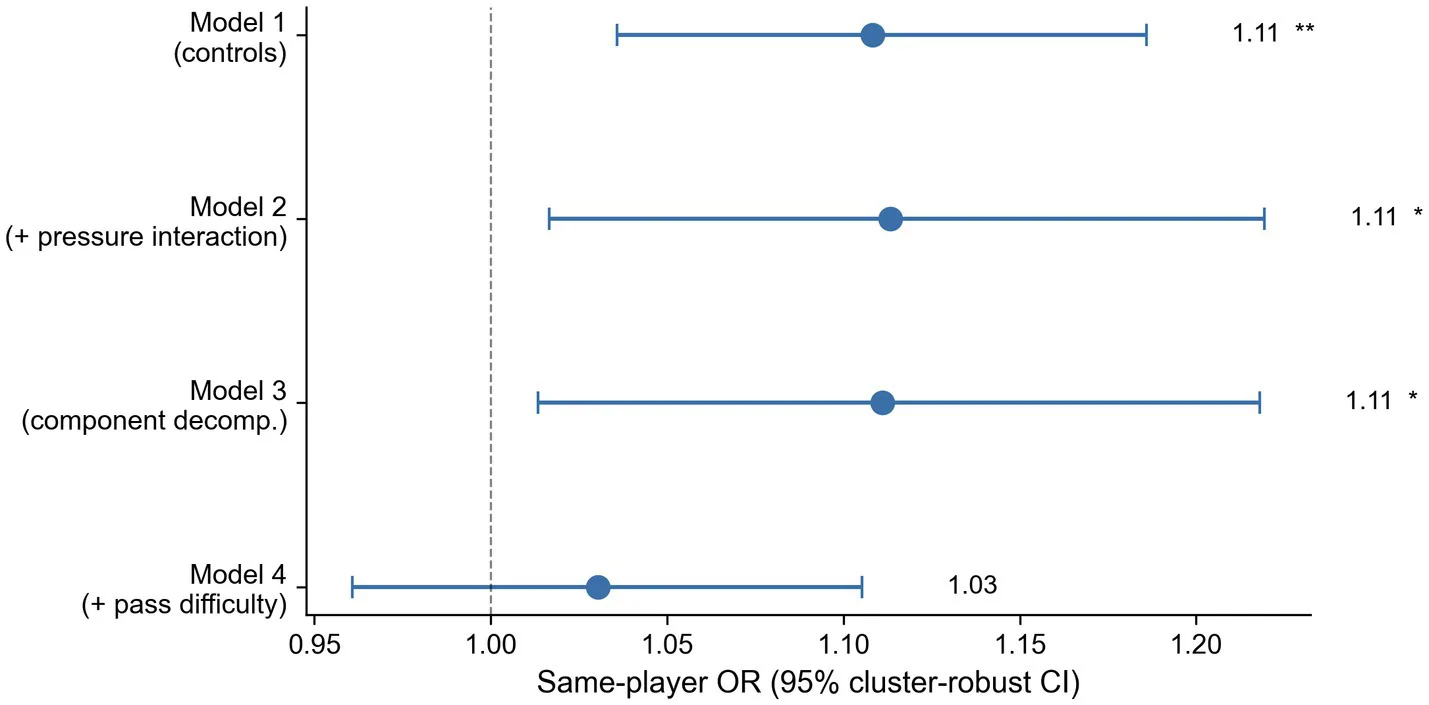
Adjusted same-player odds ratios with 95% two-way cluster-robust confidence intervals across the four pre-specified logistic regressions. The dashed line at OR = 1 indicates no same-player effect. Model 1 includes the pressure index and position fixed effects; Model 2 adds the same-player × pressure-index interaction; Model 3 replaces the composite index with its four components and adds four corresponding same-player × component interactions; Model 4 adds the three pass-difficulty covariates (log pass length, forward-pass indicator, three-level pass height) to the Model 1 specification. Standard errors are two-way cluster-robust, clustered on the player taking the follow-up pass and on match.

The component-level pressure findings in Model 3 are broadly consistent with Study 1. Late in a balanced match (closing in balance) raised follow-up error probability (*OR* = 1.25, 95% CI [1.16, 1.34], *p* < 0.001), as did knockout-fixture status (*OR* = 1.16, 95% CI [1.03, 1.31], *p* = 0.018). Trailing on the scoreboard (*OR* = 0.97, 95% CI [0.89, 1.06], *p* = 0.497) and numerical disadvantage (*OR* = 1.11, 95% CI [0.93, 1.33], *p* = 0.248) showed no reliable association with follow-up error probability. Coefficient tables for all four models are provided in Supplementary Tables S4–S7.

#### Discussion

Study 2 provides evidence of interpersonal error elevation in professional football: following an unforced pass error, teammates who take the follow-up pass perform worse than their own baseline error rate by +3.93 pp. The residual contagion test isolates this elevation from individual differences in which players take the follow-up pass after errors, so the elevation cannot be explained by higher-error-rate players being systematically involved in post-error sequences. The interpersonal effect is approximately half the magnitude of the within-person compounding effect documented in Study 1 (44%), which implies an absolute elevation of about 8 pp. following a player’s own prior error. The two within-player measures used across Studies 1 and 2 are not directly equivalent, however. Study 1’s prior-error indicator captures the player’s next unforced pass at any subsequent point in the match, regardless of intervening teammate actions. Study 2’s same-player follow-up captures only the narrower subset of cases in which the source player is also the team’s immediate next passer (typically quick recoveries).

The findings of Study 2 add further support to the qualitative research of Wergin and colleagues on collective team collapses (e.g., Wergin et al., 2018, 2025). Wergin et al. (2018) reported that unforced error accumulation was a common factor preceding a critical team collapse: Athlete 8 (field hockey), for example, described how the bad performance of individual players caused other players of the team to fail as well: *“The collapse was triggered by individual mistakes and that led to collective failure. I would say that the individual mistakes caused an insecurity in the whole team and mistakes increased even more”* (Wergin et al., 2018, p. 10). Another critical *other*-referenced event identified by Wergin et al. (2018) was an opponent scoring. Recent archival analyses of penalty shootout data suggest that opponent performance may be a more powerful driver of probability-of-failure expectations than teammate performance (Boto-García and Bucciol, 2025). The probability of scoring was 17.9 percentage points lower after the opposing team scored but was independent of the outcome of a teammate’s own preceding penalty kick. More research is required to further understand how social observations of opponents and teammates drive pressure contagion effects in team sports.

Taken together, Studies 1 and 2 indicate that passing error dynamics operate mainly within individuals, with a smaller component of interpersonal spread (error contagion). A consistent feature of both studies is the role of pass difficulty in predicting an error. In Study 1, pass-difficulty covariates mediated ~60% of the pressure and prior-error main effects. In Study 2, the same covariates reduced the adjusted same-player versus different-player contrast to statistical non-significance (Model 4: OR = 1.03, *p* = 0.38). Unlike Study 1, where a significant residual effect survives pass-difficulty adjustment, Study 2’s residual is indistinguishable from unity, so we cannot rule out that little or no same-player-specific effect existed independently of pass selection to begin with. Study 3 therefore returns to the within-player case, where a residual effect does survive adjustment, to ask how far pass selection carries error compounding as chains lengthen and whether it accounts for differences between players.

### Study 3: From error pairs to error chains. Do errors compound?

#### Overview and rationale

The findings from Studies 1 and 2 raise a natural follow-up question. If errors are primarily carried within the individual, what happens when they accumulate? Elite footballers describe in vivid terms how a single error can spiral into a cascade of further mistakes; a “vicious circle,” a “negative spiral,” a “vortex” (Jekauc et al., 2024b). Within ACTS, probability-of-failure appraisals are revised in light of accumulating evidence rather than reset after each action. Each consecutive error should prompt a progressively larger revision in perceived failure probability and a shift from an unstable, external attribution toward a more stable, internal one. Study 1 (as with the entire literature base) documented a single lag-1 step, while Study 2 has shown that this step’s consequences are largely retained within the person. Study 3 therefore examines what happens when errors compound across successive actions within the same player: does error probability escalate progressively (compound) with chain depth, and do players differ systematically in their susceptibility to this escalation?

An important analytical goal is to separate two sources of potential individual variation: (1) *baseline error tendency* reflects a combination of technical proficiency and risk preference (some players make more pass errors generally), whereas (2) *psychological susceptibility to chain escalation* reflects the appraisal pathway through which accumulated failure feedback revises the perceived probability of further failure (some players are disproportionately affected once errors accumulate). From an ACTS perspective a player’s general error rate need not predict how they respond once an error has occurred. ACTS focuses more on individual differences in attentional or interpretive bias for error-related stimuli in explaining resilience or vulnerability to errors (Eysenck and Wilson, 2016). However, as reflected in our discussion of Evans and Crosby’s (2021) findings, better performers may paradoxically be *more* vulnerable to chains of errors, given their relative scarcity and potentially identity-threatening nature (Musco et al., 2023; Swann, 2012).

Study 3 explores what happens when error and success chains lengthen, and tests three predictions: (1) the negative effect of errors will compound so that the effect of multiple consecutive errors will be stronger than for a single pair (“errors compound”; Wilson and Eysenck, 2025); (2) this effect will be stronger than the corresponding effect for compounding successes (“bad is stronger than good”; Baumeister et al., 2001; Elmore and Urbaczewski, 2018). (3) Players with lower baseline error tendency will be associated with greater chain susceptibility (Evans and Crosby, 2021). We also examine, exploratorily, whether this vulnerability is behaviourally expressed in pass selection, with lower-baseline players shifting further toward difficult passes as chains deepen, consistent with loss-induced risk-seeking (Thaler and Johnson, 1990) and identity protection (Oyserman, 2024).

#### Method

##### Sample and variables

Study 3 reused Study 1’s exact analytic sample of 272,381 unforced classifiable pass actions (sb_pressure = 0, is_classifiable = 1) with valid prior-action data from 330 matches and 2,202 unique players, and errors defined as before (incomplete unforced passes; pass_outcome non-null). As in Study 1, each player’s first unforced pass per match was excluded as lacking a valid prior. Because Study 1 and Study 3 draw on identical underlying passes, their findings should not be read as independent replications of one another.

Two within-player sequential variables were constructed from this sample. Error-chain depth indexed the number of consecutive immediately preceding unforced pass *errors* by the same player in the same match. Depth 0 meant the player’s previous unforced pass was successful (i.e., the current pass follows a success, regardless of whether the current pass itself succeeds or errs); depth 1 indicated one consecutive prior error; depth 2 indicated two; and so on. Because chain depth is a within-player, within-match measure, any successful action resets the player to depth 0. Success-chain depth was constructed symmetrically: Depth 0 meant the player’s previous unforced pass was an error, and any error reset depth to 0.

Both variables were capped at 6 for modelling. On the error side this threshold excluded negligible information (depth ≥ 6 comprised only 0.10% of the sample; Supplementary Table S8). On the success side, however, streaks of 6 or more passes were common (30.8% of the sample; Supplementary Table S8b), so the same cap is considerably more truncating. The consequences of this asymmetry for the success-chain estimates are examined in a sensitivity analysis (Supplementary Tables S8c,d—see Results).

##### Analytical strategy

The primary analyses were binomial generalised linear mixed models (GLMMs) with random intercepts (per player and per match) and a per-player random slope for the relevant chain depth^7^. Models were fitted in R using lme4 (Bates et al., 2015) with the default Laplace approximation and the bobyqa optimiser. All other analyses (descriptives, individual-differences correlations, sensitivity checks) used Python’s statsmodels (Seabold and Perktold, 2010). The decision to fit the multilevel models in R reflects the absence of a reliable Python implementation of binomial GLMMs with random slopes at this sample size.

Four models were fitted in parallel. *Model 1 (error)* regressed the binary error outcome on error chain depth and seven-category position. *Model 2 (error)* added the three pass-difficulty covariates from Studies 1 and 2 to test how much of the chain effect operates through what is attempted as chains deepen versus through degraded execution of comparable passes. *Model 1 (success)* and *Model 2 (success)* applied the identical structure to the success-chain side (outcome is_success, predictor success_chain_depth). For each side, a likelihood-ratio test (LRT) compared Model 1 against a random-intercepts-only specification (the same fixed-effect structure, but without the per-player random slope). A significant LRT establishes that players reliably differ in chain susceptibility independently of their baseline tendency.

To characterise individual differences in chain susceptibility, empirical-Bayes Best Linear Unbiased Predictors (BLUPs) of the random effects were extracted from Model 1 separately for each side.^8^ The primary individual-differences statistic was the Spearman rank correlation between each player’s intercept BLUP (baseline error or success tendency within position) and their slope BLUP (chain susceptibility). Individual estimates were restricted to players with at least 10 observations at chain depth ≥ 1 (*n* = 1,289). This threshold balances estimation reliability against sample retention: mean BLUP slope standard error was 0.0822 in the lowest-observation tertile, 0.0796 in the middle tertile, and 0.0742 in the highest tertile, a modest, gradual decline rather than a sharp cliff at the threshold, supporting ≥10 as a reasonable cut rather than an arbitrary one. Predicted probabilities plotted in Figure 5 include each player’s and match’s estimated random effects (BLUPs), so the predicted line closely tracks observed rates at every depth. A small residual gap remains at the deepest error-chain bin (depth 6), consistent with the small, non-random subset of players who reach that depth.

**Figure 5:**
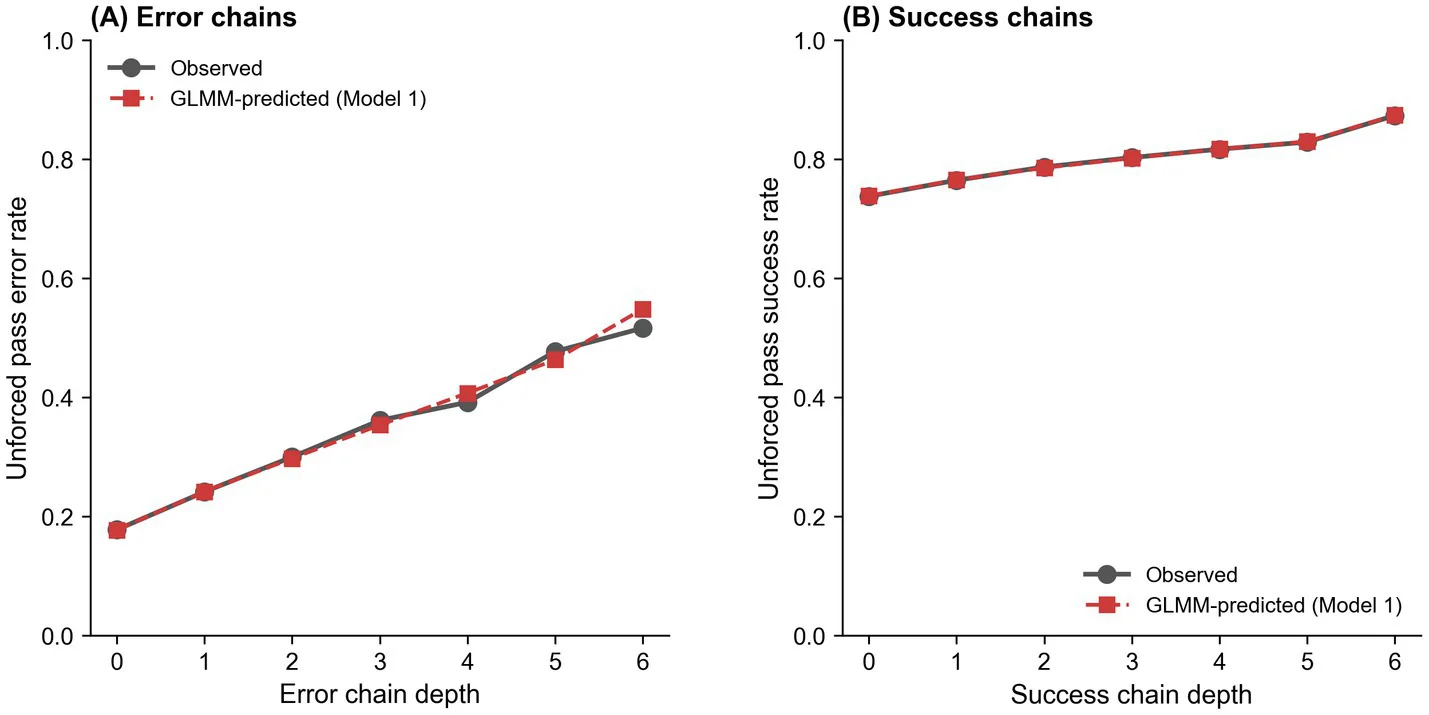
Error chain and success chain dynamics. **(A)** Probability of an unforced pass error as a function of error chain depth (consecutive prior unforced pass errors by the same player in the same match). **(B)** Probability of an unforced pass success as a function of success chain depth (consecutive prior unforced pass successes by the same player in the same match). Dark grey lines with circular markers show observed proportions, and red dashed lines with square markers show Model 1 predicted probabilities incorporating each player’s and match’s estimated random effects (BLUPs). The predicted line tracks observed proportions closely at every depth, with a small residual gap at the deepest error-chain bin (depth 6). *N* = 272,381 unforced passes from 330 matches and 2,202 players. Chain depths capped at 6. Both error and success sides show monotonic escalation, but the absolute swing is approximately threefold on the error side (17.7% → 51.6% across depths 0–6) versus less than 15 percentage points on the success side (73.8% → 87.3%).

#### Results

##### Population-average compounding

Observed unforced-pass error rates climbed monotonically with error chain depth (Supplementary Table S8), from 17.7% at depth 0 to 51.6% at depth 6, an absolute increase of 33.9 percentage points with no plateau across the observable range. Players observed at the deepest bins are not a random sample of those at depth 0, as by definition they are the players who produced six consecutive errors. The curve is therefore partly a selection effect. Model 1 confirmed a statistically significant population-average chain effect (OR = 1.066 per consecutive prior error, 95% CI [1.050, 1.083], *p* < 0.001; Supplementary Table S9). The random-slope structure significantly improved fit over an intercepts-only specification (LRT χ^2^(2) ≈ 52.3, *p* < 0.001), confirming that players differ reliably in chain susceptibility independently of their baseline error tendency. A categorical specification of chain depth did not improve fit (LRT χ^2^(5) = 2.57, *p* = 0.766; AIC: linear = 251929.5, categorical = 251936.9), supporting the linear form used throughout.

Adjusting for pass-difficulty (Model 2) reduced the population-average chain slope, with the OR dropping from 1.066 to 1.017 (95% CI [0.999, 1.035], *p* = 0.065). Applying the same KHB decomposition used in Study 1 to correct for logit-rescaling (Karlson et al., 2012), pass selection mediated 91.7% of the population-average error-compounding effect. At the population level, compounding operates through what players choose to attempt deeper in a chain (pass selection), rather than through degraded execution of comparable passes. Critically, the same adjustment left the between-player variation in chain susceptibility essentially unchanged (random-slope SD = 0.093 in Model 1, 0.095 in Model 2; Supplementary Table S10), indicating that pass difficulty does not account for *individual* differences in chain susceptibility. Figure 5A shows the observed and Model 1 predicted error rates by chain depth.

##### Asymmetry between error and success compounding

Descriptively, success-chain effects are also present in the data, rising from 73.8% at success-chain depth 0 to 87.3% at depth 6 (Figure 5B; Supplementary Table S8). Model 1 (success) returned a population-average slope of OR = 1.030 per consecutive prior success (95% CI [1.024, 1.036], *p* < 0.001), reduced to OR = 1.022 (95% CI [1.015, 1.028], *p* < 0.001) under pass-difficulty adjustment in Model 2 (success). KHB decomposition indicated pass selection mediates 74.6% of the success-chain effect. Pass selection is therefore a major pathway on both sides of the error/success asymmetry.

Although both errors and successes show monotonic chain effects, an asymmetry between errors and successes emerges at the *within*-player level in two specific ways. First, the between-player variation in chain susceptibility was approximately twice as large on the error side as on the success side (random-slope SD = 0.093 vs. 0.044 log-odds per step; Supplementary Table S10). Capping chains at 6 understates the true asymmetry, because the success-side estimate is truncated far more severely than the error side: 30.8% of the success-chain sample reaches depth 6, versus 0.10% on the error side (Supplementary Table S8b). Re-fitting the success-chain model at a cap of 65 (the depth at which success-side capping severity (0.09%) most closely matches the error side’s) reduces the success-side random-slope SD from 0.044 to 0.012; a ~4-fold reduction. The true asymmetry is therefore closer to eight-fold than two-fold (Supplementary Tables S8c,d). Second, the relationship between a player’s baseline tendency and their chain susceptibility was strong and negative for errors but essentially absent for successes (see next section).

##### Individual differences in error chain susceptibility

Empirical-Bayes random effects from Model 1 (error) were extracted to characterise each player’s joint position on baseline error tendency (random *intercept*) and error chain susceptibility (random *slope*). The model-implied correlation between these two random effects (computed from the covariance matrix) was *r* = −0.599, indicating that players with lower baseline error rates had steeper error chain slopes (visible in the clear diagonal plot in Figure 6). The empirical Spearman correlation between the extracted BLUPs in the primary sample (≥ 10 observations at chain depth ≥ 1) was *r* = −0.91, *p* < 0.001, *n* = 1,289. Figure 6 plots the joint distribution of Model 1 error random intercept and random slope, stratified by observation tier (how many times that player was observed at error-chain depth ≥ 1).

**Figure 6:**
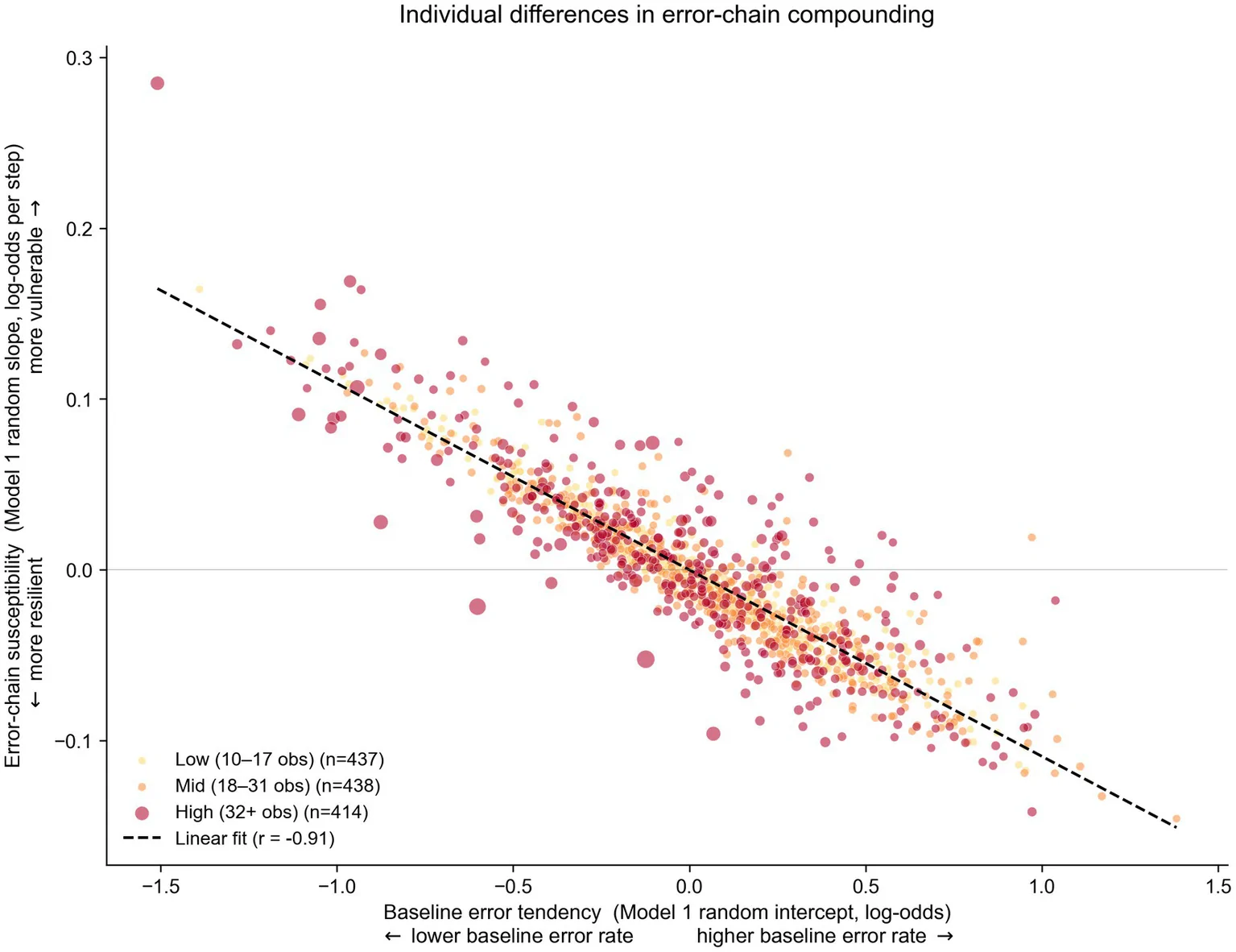
Individual differences in error chain compounding. Each point represents one of *n* = 1,289 players meeting the inclusion threshold (≥ 10 unforced passes at error chain depth ≥ 1). X-axis: Model 1 random intercept BLUP (baseline error tendency within position, log-odds). Y-axis: Model 1 random slope BLUP (error chain susceptibility, log-odds per step). Point colour denotes observation tier (tertiles of within-chain observations); point size scales with within-chain observations. Dashed line is an OLS fit through the BLUPs.

All nine fitted GLMMs converged with no singular-fit warnings^9^. Because BLUPs are shrunk toward a population-level covariance structure that already encodes an intercept–slope correlation, the BLUP-based correlation is not a fully independent check on this parameter. We therefore also fitted separate per-player logistic regressions with no shared random-effects structure, for the 465 players with ≥ 30 observations at chain depth ≥ 1, correlating each player’s unpooled slope against their observed baseline error rate: r = −0.270 (Spearman, *p* < 0.001). The unpooled correlation is smaller than the BLUP-based estimate, but runs in the same direction, confirming the relationship is not purely a shrinkage artefact. A sensitivity sweep across inclusion thresholds from ≥ 5 to ≥ 100 observations (Supplementary Table S11; Supplementary Figure S2) shows the BLUP-based correlation weakening as shrinkage is reduced, from r = −0.92 (*n* = 1,685) at the most inclusive threshold to r = −0.79 to −0.81 (*n* = 59–96) at the most stringent; revealing a more modest effect than the primary estimate alone suggests. The same negative correlation held within every one of the seven position categories (Spearman *r* between −0.855 [striker] and −0.953 [centre-back]; Supplementary Table S12). The within-player association between baseline error tendency and chain susceptibility is therefore robust across positions and not an artefact of either shrinkage or position composition.

The parallel correlation on the success-chain side (Supplementary Table S11, same sweep) was small but statistically reliable across most of the range tested, from *r* = −0.12 (*n* = 1,685) at the most inclusive threshold to *r* = −0.16 (*n* = 465) at the most stringent threshold (all *p* ≤ 0.001). Significance was lost only once the sample became sparse (*n* ≤ 191; *p* = 0.072–0.472). These correlations remained an order of magnitude smaller than the corresponding error-side correlation at every threshold (Supplementary Figure S2). The asymmetry between errors and successes is therefore most sharply expressed at the individual-differences level.

##### Pass selection covariates

Because baseline error tendency is estimated without adjustment for what players attempt, it indexes pass selection as well as execution. Re-estimating both random effects with the pass-difficulty covariates included (Model 2) reduced the between-player variance in baseline error tendency by 45% (SD = 0.480 to 0.357) while leaving variance in chain susceptibility unchanged (SD = 0.093 to 0.095). Including the pass difficulty covariates also halved the association between baseline error tendency and chain susceptibility (model-implied r = −0.599 to −0.315; BLUP r = −0.908 to −0.578), a 60% reduction in their covariance. Pass selection therefore carries most of the error tendency-escalation association and about half the variation in baseline error tendency, without entirely accounting for either. Because Model 2 adjusts for covariates that mediate most of the chain effect, these estimates are net of observable pass difficulty rather than a causal decomposition. All correlation estimates for this relationship are collated in Supplementary Table S15.

To descriptively compare changes in these pass selection effects across chains, players in the primary sample were stratified into tertiles on Model 1 intercept BLUP. This yielded three groups labelled “low-error” (most negative intercepts, lowest baseline error tendency within position), “mid-error,” and “high-error” (most positive intercepts, highest baseline error tendency within position). For each combination of tertile and error chain depth (0 through 6), the three pass-difficulty variables were computed as descriptive means with standard errors. All three pass-difficulty variables show a similar pattern (see Figure 7). First, at depth 0, the low-error group favoured less difficult (shorter, more sideways, more grounded) passes than both more error-prone tertiles. Second, all three tertiles then shifted toward longer, more forward, more aerial passes as chains deepened, converging by depth 6. However, the low-error tertile shifted further, most clearly on aerial-pass rate, where the shift was roughly double the high-error tertile’s (+53 pp. vs. +26.5 pp) despite similar endpoints. Descriptive values for each tertile × depth cell are tabulated in Supplementary Table S13 and exploratory analyses of slope differences across tertiles are reported in Supplementary Table S14.

**Figure 7:**
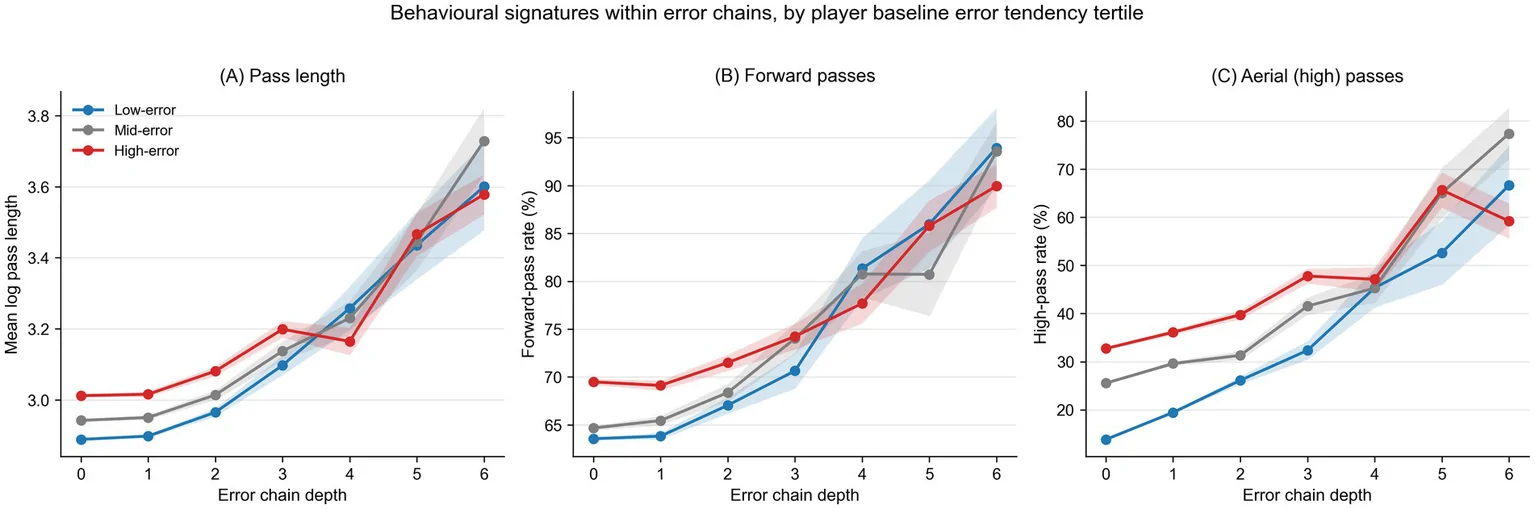
Behavioural signatures within error chains, by player baseline error tertile. Players meeting the primary inclusion threshold (*n* = 1,289) were stratified into tertiles on Model 1 random intercept BLUP: “low-error” (*n* = 430, lowest baseline error tendency within position), “mid-error” (*n* = 429), and “high-error” (*n* = 430, highest). For each tertile, three pass-difficulty variables are plotted as functions of error chain depth: **(A)** mean log pass length, **(B)** forward-pass rate, and **(C)** high-pass (aerial) rate. Shaded bands show ±1 SE. All three tertiles shift toward longer, more forward, and more aerial passes as chains deepen, but the low-error tertile shifts most dramatically, converging with the high-error tertile by depth 6. This tertile difference in slope was only statistically significant for the aerial-pass rate (Panel C; interaction *p* < 0.001).

#### Discussion

Study 3 produced four interconnected findings. First, unforced pass errors compound at the population level: error probability climbed from 17.7% at depth 0 to 51.6% at depth 6, with Model 1 returning *OR* = 1.066 per consecutive prior error. Second, approximately 92% of this population-average compounding operates through pass selection rather than degraded execution of comparable passes; players reach for longer, more forward, and more aerial passes as chains deepen. Third, the proposed asymmetry between error and success compounding (e.g., Elmore and Urbaczewski, 2018) was supported in the examination of individual differences, with error chains carrying approximately eight times the between-player variation of success chains. Fourth, the players whose baseline error rates were lowest within their position showed the steepest error chain slopes (cf. Evans and Crosby, 2021); an effect that was present within every position group (Supplementary Table S12) and was behaviourally indexed via dramatic shifts in pass selection across error chains (Figure 7).

As noted above, the compounding curve is partly a selection effect. What is not explained by selection is that the escalation is accompanied by a change in the riskiness and difficulty of pass selection. We have suggested a schema incongruence interpretation (Swann, 2012) for why players with lower baseline error rates were more likely to be disrupted by chains of errors. Compounding errors may disrupt decision-making goal setting as well as the attentional control underlying option generation and execution (Eysenck and Wilson, 2016, see General Discussion for more). Less error-prone players may choose to attempt high-value “redemptive” actions—more forward, aerial, and longer passes—to restore their threatened sense of competence following compounding errors (Oyserman, 2024).

Such a redemptive-action account mirrors a well-established asymmetry in the self-regulation literature. Under ego threat, individuals with higher self-esteem or more invested status set inappropriately risky goals, often performing worse than those with less at stake who remain conservative (Baumeister et al., 1993). Elevated risk-taking following a negative outcome has also been documented in financial decision-making (Imas, 2016) and under induced anger (Lerner et al., 2015), though these remain analogies from other domains rather than direct tests of the mechanism proposed here. The move towards increasingly high value passes as errors compound may also reflect a more general action bias (Patt and Zeckhauser, 2000). Across decision domains, failures of inaction are judged and felt more harshly than equivalent failures of action, biasing decision-makers toward visible intervention even when it is not the higher-expected-value choice (Patt and Zeckhauser, 2000). This pattern has been documented directly in football goalkeepers’ penalty-kick decisions (Bar-Eli et al., 2007) and supports our observations for why passing choices might become more spectacular after compounding errors.

## General discussion

The current paper reports three studies that make use of a large set of real-world performance data to address theoretically driven research questions which have important, applied implications. While the research builds on previous work (e.g., Harris et al., 2019, 2021) it creates new knowledge on the potential psychological impacts of errors on inter- and intra-individual error compounding. First, it shows that errors are not just individually disruptive (Study 1), they are also contagious. While making an error raises a player’s own next-action error rate by 8 percentage points, a teammate’s error rate is still almost 4 percentage points above their own personal norm (Study 2). Most importantly, there are individual variations in how the impact of successive errors might compound, which affect decision-making and execution elements of passing performance in a way that does not occur for compounded successes. Players with the lowest baseline error tendency appear most vulnerable to compounded error effects (Study 3).

One of the aims of the study was to seek novel ways to explore psychological mechanisms through performance data. We recognise that these observational StatsBomb data do not provide access to players’ internal threat appraisals, so our interpretation of a psychological explanation for behavioural effects can be challenged. For example, part of the post-error and pressure elevation may reflect tactical deterioration, rather than psychological carryover. However, we know from recent qualitative research with elite football players that cascading cycles of anxiety, rumination, self-focus, and declining confidence do follow high-profile mistakes (Jekauc et al., 2024b). The emotive terms that Jekauc’s participants used to describe the impact of such errors reinforces our interpretation that there is a strong psychological component as proposed in ACTS (Eysenck and Wilson, 2016).

Using similar methodology to Evans and Crosby (2021) we also found that better performers were paradoxically *more* vulnerable to chains of errors. We proposed that error chains create a larger expectancy violation and a more self-discrepant event for players whose baseline error rate is low (Musco et al., 2023; Swann, 2012). For such a competitor, an error likely creates an immediate, painful gap between their *actual* performance and their internalised *ideal* or *ought* self-guides (being flawless and reliable; Higgins, 1987). The psychological discomfort may explain two effects seen in our data. First, there is the proactive move towards more risky and high value actions as outlined in our redemptive-action postulation (Study 3 Discussion; see also Bar-Eli et al., 2007; Oyserman, 2024). Second, pass execution may be impaired by what Kuhl (1984) terms a shift from action orientation toward a state orientation. Instead of focusing on the next pass, the player may become cognitively locked into a state of retrospective rumination over the failure and its threat to their self-concept. Impaired attention was explicitly referred to as key consequence of team errors in Wergin et al.’s (2018) qualitative study of decreased performance contagion and is aligned with the attentional disruption mechanism proposed in ACT (Eysenck et al., 2007). Both attentional (execution) and goal-selection (pass selection) effects could be framed as expressions of the same underlying disruption—failure to disengage from the prior error—but manifesting differently. Our research design does not provide the ability to interrogate the relative balance of such arguments further. Indeed, our data contain no measure of players’ appraisals, so any psychological interpretation remains one among several we cannot separate. These include opponents pressing reliable passers harder after a visible error, or teams shifting tactical approach after losing possession.

Future research should therefore explore additional individual differences that might explain psychological resilience to error compounding. ACTS predicts that attentional and interpretive biases toward error-related stimuli should shape such resilience (Eysenck and Wilson, 2016). We termed this tendency error amnesia (Harris et al., 2019), and later defined it as, “the capacity to rapidly disengage from a prior mistake with minimal emotional and attentional carryover” (Wilson and Eysenck, 2025). Error amnesia has not yet been investigated empirically, though an unvalidated scale is in development (Park and Wilson, 2026). Related constructs may help anchor this work both conceptually and psychometrically. Intentional forgetting, the suppression of unwanted thoughts to preserve limited-capacity cognitive resources (Fawcett and Hulbert, 2020), is one candidate. While emotionally charged memories have been shown to be harder to suppress (Hall et al., 2021), athletes higher in mental toughness show greater capacity for such intentional forgetting (Dewhurst et al., 2019). Mental toughness, and potentially other trait-level resilience constructs, may therefore share conceptual ground with error amnesia. Testing how these constructs converge, diverge, or independently predict resistance to error chains would extend the present findings and help clarify which psychological profiles leave athletes most vulnerable to compounding errors.

### Implications

A central implication of these data is that the cost of an error lies not in the error itself, but in what it becomes if allowed to compound. Errors are unavoidable in sport and in life, and so the solution cannot be to prevent them (Rotella, 1995). We would therefore agree with Jekauc et al.’s (2024b) recommendations that sport psychologists should work with athletes to help them understand how negative events can trigger a chain reaction that leads to further negative outcomes, and develop a more proactive approach to managing them. Our data point to four starting points: (1) containing identity threat, (2) breaking the chain early, (3) limiting interpersonal contagion, and (4) profiling vulnerability. None of the approaches below have been tested against the specific mechanism identified here (riskier pass selection following an error), so they are plausible extrapolations from adjacent evidence rather than validated interventions for error compounding.

First, self-compassion-based approaches may be especially useful in containing the identity threat. Brief interventions can reduce self-criticism, rumination, and concern over mistakes in highly self-critical athletes without encouraging complacency (Mosewich et al., 2013), and mindfulness- and acceptance-based work similarly fosters a non-judgemental relationship with error-related thoughts rather than their suppression (Gardner and Moore, 2007). Second, post-mistake routines that pair an attentional or physical anchor with a deliberate refocusing cue (Ravizza, 2006) offer a practical means of re-establishing task focus. Third, limiting interpersonal contagion recognises that a performer’s visible reaction to an error is itself an input to teammates’ subsequent performance (Wergin et al., 2018, 2025). Athlete leaders modelling composed recovery may thus act as a circuit breaker for the wider team (Fransen et al., 2015). Finally, profiling vulnerability acknowledges that these needs are not uniform. A validated measure of error recovery could help practitioners direct the right intervention to the right athlete and prioritise it for the contexts in which compounding is most pronounced (Park and Wilson, 2026).

### Limitations

A strength of the research is that we are testing theoretically derived hypotheses using real-world, open data that is available to everyone. As we have also made our analyses code freely accessible^10^ we welcome other researchers confirming and building on our findings to address additional questions. The work has two related limitations. First, we infer psychological states from performance data rather than measuring them directly. Qualitative approaches can access the athlete’s own account of an error and its aftermath in a way behavioural data cannot (e.g., Jekauc et al., 2024b; Wergin et al., 2018). Experimental designs that manipulate errors and pressure while recording self-report and psychophysiological metrics could establish the causal mechanisms that observational data can only imply (e.g., Harris et al., 2023). Caso et al. (2026) note that behavioural observation retains the advantage of real-world ecological validity that both qualitative and experimental approaches sacrifice to some degree. Triangulating across all three approaches would strengthen confidence in the psychological mechanisms we propose.

Second, our behavioural measures were themselves relatively coarse. Access to more detailed, paid datasets (e.g., Opta [Stats Perform], Wyscout [Hudl]) and video-based analyses could support examination of finer-grained behaviours and emotional responses than the present dataset allows (see recent review by Caso et al., 2026). Recent studies illustrate the potential opportunities here. Jekauc et al. (2024a) trained AI models to recognise negative affective states from tennis players’ facial expressions during live competitive data. Lu and Zhong (2023) used high-frame-rate, contactless cameras to track archers’ heart rate during the 2020 Olympic archery competition. Of specific interest to the predictions of ACTS, Lu and Zhong found that a poor result on the previous shot elevated subsequent heart rate and raised the probability of a subsequent failure, an effect most pronounced when shooting second or later in the match. Such multimodal approaches, made possible by technological advances, provide exciting opportunities for studying the psychological impacts of compounding errors from real-world settings.

A further limitation was that we did not replicate the significant pressure × error interaction found in our previous studies (Harris et al., 2019, 2021). Both pressure and prior error independently increased the odds of a subsequent error, but the interaction was reversed. This does not necessarily contradict ACTS but may suggest that situational pressure and the accumulation of failure operate as psychologically distinct pathways rather than combining multiplicatively (see also Klingner et al., 2023). A related limitation concerns the pressure index itself. Our focus on passing errors across all match phases may capture lower-stakes scenarios than those in the literature that informed the index’s construction. The trailing component, for instance, was included primarily on the strength of Indaco and Teqja’s (2025) finding that players underperform in attacking situations relative to expected goals when trailing. It is possible that trailing’s effect on passing behaviour specifically is weaker, or absent, compared to its effect on shooting performance.

More broadly, over 90% of passes in our data occurred at pressure levels 0 or 1 on our 4-point pressure index. This skew may explain why existing football pressure research has instead concentrated on discrete, highly leveraged individual events such as penalty shootouts, which guarantee the pressure variance that open-play passing may not provide (e.g., Jordet, 2009; Santos, 2024). From an applied standpoint, the variability in specific pressure component effects does reinforce the idea that “pressure” should not be treated as a single quantity bearing uniformly on performance, but as a composite of distinct situational triggers whose effects should be identified individually (Henderson et al., 2024).

## Conclusion

To conclude, this research provides the first application of ACTS to large-scale football event data and refines what we know about error-compounding in sport. First, errors in football are sequentially dependent: a prior error raises the probability of the next, and this within-player compounding deepens monotonically across chains of consecutive failure rather than resetting after each mistake. Second, errors do not stop at the player who makes them and are somewhat contagious to teammates. Third, vulnerability to error compounding varies between players, and those with the lowest baseline error rates showed the steepest escalation, an effect that appeared to be influenced by both attentional disruption to pass execution and a goal-selection disruption to pass selection. Across all three studies, what a player attempted changed as failure accumulated: what compounds is not the error alone but what a player reaches for next. The practical implication is that interventions should perhaps target the accumulation of failure feedback specifically, and should be tailored to a player’s vulnerability profile: a low error rate is no guarantee of resilience to a chain of errors.

## Data Availability

The event data analysed in this study were provided by StatsBomb (statsbomb.com) under their open data license for non-commercial research use and are publicly available at https://github.com/statsbomb/open-data. The processed datasets, analysis code, and full output tables generated for this study are available in the OSF repository at https://osf.io/52xwc/files/osfstorage.

## Footnotes

1 We classified 7 positions from StatsBomb definitions: Goalkeeper, Centre Back, Full Back (includes wing-back variants), Defensive Midfield, Central Midfield (central/wide), Attacking Midfielder/Winger, Striker. Because position reflects StatsBomb’s per-action tactical tagging rather than a fixed player attribute, some players (particularly rotation and squad players) are tagged with more than one category across the sample. There is not space to go into the separate effects in this manuscript, however, primary conclusions were not affected by positional effects. A brief overview of differences between positions for each study is provided in the Supplementary Materials.

2 As well as a “scripts” folder containing the analysis scripts, https://osf.io/52xwc/files/osfstorage also includes a README file to explain how to run the study; a “data” folder (with the processed dataset); a “docs” folder including this manuscript and Supplementary files; and an “output” folder with .csv files and figures.

3 For illustration, the top 4 occurring events were (N/% of the sample): Pass (334,181/28%), Ball Receipt (310,703/26%), Carry (268,886/23%), Pressure (107,944/9%). Note that in StatsBomb terminology “under_pressure” is a specific, event-level metric that captures when a defensive player closes down an opponent in possession of the ball to limit their time and space, typically within a 4–5 yard radius.

4 This threshold was applied uniformly regardless of whether a match subsequently proceeded to extra time, which only occurred in 8 of the 330 matches (2.4%). Because players cannot anticipate extra time in advance, minute-75 in a match that is ultimately drawn after 90 min should still carry elevated stakes at the time the action occurs.

5 We did not cluster on competition as there were too few for reliable inference (*n* = 10). Match-level clustering (330 clusters) is well identified and mirrors the match random intercept used in Study 3.

6 Because cost-of-failure situations might be disproportionately concentrated in a team’s defensive third, we additionally tested whether the pressure × prior-error interaction was robust to controlling for this potential “location” confounder (Supplementary Table S1, Specification C).

7 This is conceptually similar to the logistic random-effect models used by Elmore and Urbaczewski (2018) in golf. They fitted random-intercept-plus-random-slope logistic models with player-specific random effects, controlling for hole difficulty (field average) and par type.

8 BLUPs are posterior modes of each player’s random intercept and random slope, given the fitted model and that player’s own observed data. Empirical-Bayes estimation shrinks each player’s estimates toward the population mean of the random-effects distribution, so players with many in-chain observations are estimated essentially from their own data, while players with few in-chain observations are pulled toward the population regression line. The empirical correlation between extracted BLUPs is therefore typically larger in absolute magnitude than the model-implied correlation taken from the random-effects covariance matrix. Both quantities are reported.

9 The nine models reflect a primary and a pass-difficulty-adjusted specification for each side, an intercept-only likelihood-ratio baseline for each side, and three further models testing linearity and capping-severity sensitivity.

10
https://osf.io/52xwc/files/osfstorage
