# Supplementary material for: When, where, and for whom do errors compound? Error sequences, contagion, and individual vulnerability in professional football
Source: Front Psychol. 2026 Oct 26;17:1945783. doi: 10.3389/fpsyg.2026.1945783 (PMC13745248; doi:10.3389/fpsyg.2026.1945783)
Supplement: Supplementary file 1 [file Supplementary_file_1.DOCX]

**Supplementary Materials**

*When, Where, and for Whom Do Errors Compound? Error Sequences, Contagion, and Individual Vulnerability in Professional Football*

**Study 1 Supplementary Materials**

***Table S1.*** *Robustness of the pressure × prior-error interaction across alternative model specifications.*

| **Specification** | ***OR*** | ***95% CI*** | ***p*** | **Sig** |
| --- | --- | --- | --- | --- |
| A. Primary specification | 0.906 | [0.865, 0.949] | < .001 | *** |
| B. + trailing held separate with own interaction | 0.959 | [0.902, 1.020] | 0.183 |  |
| C. + defensive-third location | 0.907 | [0.867, 0.950] | < .001 | *** |
| D. + trailing separate + defensive-third | 0.960 | [0.903, 1.019] | 0.181 |  |
| E. No position fixed-effect control | 0.900 | [0.852, 0.950] | < .001 | *** |
| F. Composite excludes numerical disadvantage | 0.902 | [0.859, 0.948] | < .001 | *** |

*Note.* All models are logistic regressions of unforced pass error with two-way cluster-robust standard errors, clustered on player and match. Specification A is the primary headline reported in the main text; B holds trailing as its own predictor outside the composite with its own interaction with prior error; C adds defensive-third location as a covariate; D combines B and C; E removes the position fixed-effect control; F recomputes the composite pressure index with numerical disadvantage excluded (generated separately by step1c_specF.py). *** p < .001.

***Table S2.*** *Full coefficient estimates for the primary logistic regression model (Study 1, Model 1).*

| **Predictor** | ***β*** | ***SE*** | ***OR*** | ***95% CI*** | ***p*** |
| --- | --- | --- | --- | --- | --- |
| Intercept | -1.634 | 0.062 | 0.195 | [0.173, 0.220] | < .001 *** |
| Position: AM/W (vs CM) | 0.283 | 0.059 | 1.327 | [1.182, 1.488] | < .001 *** |
| Position: CB (vs CM) | -0.403 | 0.098 | 0.669 | [0.552, 0.810] | < .001 *** |
| Position: DM (vs CM) | -0.285 | 0.100 | 0.752 | [0.619, 0.915] | .004 ** |
| Position: FB (vs CM) | 0.085 | 0.088 | 1.089 | [0.917, 1.293] | .332 |
| Position: GK (vs CM) | 0.641 | 0.188 | 1.899 | [1.313, 2.746] | < .001 *** |
| Position: ST (vs CM) | 0.392 | 0.074 | 1.480 | [1.280, 1.712] | < .001 *** |
| Pressure index | 0.190 | 0.029 | 1.209 | [1.142, 1.279] | < .001 *** |
| Prior error | 0.450 | 0.036 | 1.568 | [1.461, 1.684] | < .001 *** |
| Pressure × prior error | -0.099 | 0.024 | 0.906 | [0.865, 0.949] | < .001 *** |

*Note.* Logistic regression on the binary unforced-pass error outcome with two-way cluster-robust standard errors, clustered on player and match. Position reference category is central midfielder (CM). N = 272,381 unforced pass actions from 2,202 unique players across 330 matches.

***Table S3.*** *Full coefficient estimates for the pass-difficulty mediation model (Study 1, Model 2).*

| **Predictor** | ***β*** | ***SE*** | ***OR*** | ***95% CI*** | ***p*** |
| --- | --- | --- | --- | --- | --- |
| Intercept | -2.414 | 0.107 | 0.089 | [0.072, 0.110] | < .001 *** |
| Position: AM/W (vs CM) | 0.422 | 0.055 | 1.525 | [1.369, 1.699] | < .001 *** |
| Position: CB (vs CM) | -0.652 | 0.075 | 0.521 | [0.449, 0.604] | < .001 *** |
| Position: DM (vs CM) | -0.426 | 0.094 | 0.653 | [0.543, 0.786] | < .001 *** |
| Position: FB (vs CM) | -0.275 | 0.048 | 0.760 | [0.691, 0.835] | < .001 *** |
| Position: GK (vs CM) | -0.339 | 0.134 | 0.713 | [0.548, 0.927] | .011 * |
| Position: ST (vs CM) | 0.536 | 0.077 | 1.710 | [1.470, 1.989] | < .001 *** |
| Pressure index | 0.085 | 0.021 | 1.089 | [1.044, 1.135] | < .001 *** |
| Prior error | 0.211 | 0.029 | 1.235 | [1.167, 1.308] | < .001 *** |
| Pressure × prior error | -0.056 | 0.023 | 0.946 | [0.903, 0.990] | .017 * |
| Log pass length | -0.299 | 0.035 | 0.742 | [0.692, 0.795] | < .001 *** |
| Forward pass | 1.224 | 0.022 | 3.402 | [3.259, 3.551] | < .001 *** |

*Note.* Logistic regression as in Table S2 with three pass-difficulty covariates added: log pass length, a forward-pass indicator, and pass-height category (three levels, reference = ground pass). N = 272,381 unforced pass actions; no rows excluded for missing pass-difficulty fields in the pass-only sample. Standard errors are two-way cluster-robust, clustered on player and match.

**Supplementary Position Analysis**

***Table S3b.*** *Position-specific descriptives and within-position prior-error effects (Study 1).*

| **Position** | **n** | **Players** | **Baseline error** | **After success** | **After error** | **Gap (pp)** | **Compounding OR** |
| --- | --- | --- | --- | --- | --- | --- | --- |
| Goalkeeper | 17,776 | 160 | 0.318 | 0.260 | 0.443 | +18.26 | 2.23*** |
| Centre-back | 58,893 | 428 | 0.132 | 0.121 | 0.209 | +8.85 | 1.89*** |
| Full-back | 60,761 | 488 | 0.203 | 0.194 | 0.242 | +4.84 | 1.31*** |
| Defensive mid. | 32,639 | 381 | 0.146 | 0.137 | 0.196 | +5.89 | 1.52*** |
| Central mid. | 55,941 | 898 | 0.190 | 0.178 | 0.237 | +5.89 | 1.41*** |
| Att. mid./Winger | 25,268 | 512 | 0.240 | 0.228 | 0.279 | +5.12 | 1.30*** |
| Striker | 21,103 | 482 | 0.261 | 0.253 | 0.285 | +3.25 | 1.18** |

*Note.* Within-position descriptive statistics and prior-error compounding odds ratios. Baseline error rate is the marginal probability of an unforced pass error within position. After-success and after-error rates condition on the immediately preceding unforced pass by the same player. Compounding OR is the position-specific prior-error odds ratio extracted from a logistic regression with prior_error × position interactions (reference = CM). *** p < .001.

***Figure S1.*** *Within-position prior-error compounding effects (Study 1).*

*Note.* (A) Forest plot of the prior-error compounding odds ratio for each of the seven position categories, with 95% two-way cluster-robust confidence intervals, from a logistic regression with prior_error × position interactions on the Study 1 primary sample. The dashed vertical line at OR = 1 marks no effect. All seven positions show statistically reliable compounding, but the magnitude varies almost two-fold from striker (OR = 1.18) to goalkeeper (OR = 2.23); in excess-odds terms the spread is roughly seven-fold. Values are tabulated in Table S3b. (B) Observed unforced pass error rates by position, split by whether the immediately preceding unforced pass by the same player in the same match was a success (white) or an error (red). Bars are unweighted descriptive proportions on the Study 1 primary sample.

**Study 2 Supplementary Materials**

***Table S4.*** *Study 2 Model 1: Same-player effect with pressure index and position controls.*

| **Predictor** | ***β*** | ***SE*** | ***OR*** | ***95% CI*** | ***p*** |
| --- | --- | --- | --- | --- | --- |
| Intercept | -1.043 | 0.041 | 0.352 | [0.325, 0.382] | < .001 *** |
| Position: AM/W (vs CM) | 0.141 | 0.052 | 1.152 | [1.041, 1.275] | .006 ** |
| Position: CB (vs CM) | -0.042 | 0.064 | 0.959 | [0.846, 1.087] | .510 |
| Position: DM (vs CM) | -0.082 | 0.073 | 0.922 | [0.799, 1.063] | .262 |
| Position: FB (vs CM) | -0.179 | 0.079 | 0.836 | [0.716, 0.976] | .023 * |
| Position: GK (vs CM) | 0.423 | 0.159 | 1.527 | [1.119, 2.084] | .008 ** |
| Position: ST (vs CM) | -0.046 | 0.073 | 0.955 | [0.828, 1.103] | .532 |
| Same player | 0.103 | 0.035 | 1.108 | [1.036, 1.186] | .003 ** |
| Pressure index | 0.087 | 0.029 | 1.091 | [1.031, 1.154] | .003 ** |

*Note.* Logistic regression on the binary follow-up-pass error outcome with two-way cluster-robust standard errors, clustered on the player taking the follow-up pass and match. Position reference category is central midfielder (CM). N = 54,409 source-error → follow-up pairs.

***Table S5.*** *Study 2 Model 2: Same-player × pressure interaction.*

| **Predictor** | ***β*** | ***SE*** | ***OR*** | ***95% CI*** | ***p*** |
| --- | --- | --- | --- | --- | --- |
| Intercept | -1.044 | 0.041 | 0.352 | [0.325, 0.382] | < .001 *** |
| Position: AM/W (vs CM) | 0.141 | 0.052 | 1.152 | [1.041, 1.275] | .006 ** |
| Position: CB (vs CM) | -0.042 | 0.064 | 0.959 | [0.846, 1.087] | .509 |
| Position: DM (vs CM) | -0.081 | 0.073 | 0.922 | [0.799, 1.063] | .263 |
| Position: FB (vs CM) | -0.179 | 0.079 | 0.836 | [0.716, 0.976] | .024 * |
| Position: GK (vs CM) | 0.423 | 0.159 | 1.527 | [1.119, 2.083] | .008 ** |
| Position: ST (vs CM) | -0.046 | 0.073 | 0.955 | [0.828, 1.102] | .531 |
| Same player | 0.107 | 0.046 | 1.113 | [1.017, 1.219] | .021 * |
| Pressure index | 0.088 | 0.030 | 1.092 | [1.030, 1.158] | .003 ** |
| Same player × pressure | -0.008 | 0.046 | 0.992 | [0.906, 1.085] | .855 |

*Note.* As Table S4 with the same-player × pressure interaction term added. The interaction is null (p = .766), indicating that situational pressure does not moderate the same-player effect. Standard errors are two-way cluster-robust, clustered on the player taking the follow-up pass and match.

***Table S6.*** *Study 2 Model 3: Pressure-component decomposition with same-player × component interactions.*

| **Predictor** | ***β*** | ***SE*** | ***OR*** | ***95% CI*** | ***p*** |
| --- | --- | --- | --- | --- | --- |
| Intercept | -1.035 | 0.042 | 0.355 | [0.327, 0.386] | < .001 *** |
| Position: AM/W (vs CM) | 0.139 | 0.052 | 1.149 | [1.038, 1.272] | .007 ** |
| Position: CB (vs CM) | -0.042 | 0.065 | 0.959 | [0.845, 1.089] | .522 |
| Position: DM (vs CM) | -0.080 | 0.073 | 0.923 | [0.800, 1.066] | .278 |
| Position: FB (vs CM) | -0.180 | 0.080 | 0.835 | [0.713, 0.977] | .025 * |
| Position: GK (vs CM) | 0.419 | 0.160 | 1.521 | [1.112, 2.082] | .009 ** |
| Position: ST (vs CM) | -0.040 | 0.073 | 0.961 | [0.833, 1.108] | .583 |
| Same player | 0.105 | 0.047 | 1.111 | [1.013, 1.218] | .025 * |
| Trailing | -0.030 | 0.044 | 0.971 | [0.891, 1.057] | .497 |
| Closing in balance | 0.219 | 0.036 | 1.245 | [1.159, 1.337] | < .001 *** |
| Knockout | 0.147 | 0.062 | 1.159 | [1.026, 1.309] | .018 * |
| Numerical disadvantage | 0.105 | 0.091 | 1.110 | [0.930, 1.326] | .248 |
| Same player × trailing | 0.013 | 0.067 | 1.013 | [0.889, 1.155] | .842 |
| Same player × closing in balance | -0.122 | 0.079 | 0.885 | [0.757, 1.034] | .123 |
| Same player × knockout | 0.060 | 0.117 | 1.062 | [0.845, 1.334] | .608 |
| Same player × numerical disadvantage | 0.158 | 0.221 | 1.171 | [0.759, 1.807] | .476 |

*Note.* Pressure-component decomposition. Two of the four pressure components return significant main effects (closing in balance and knockout); trailing and numerical disadvantage are null. None of the four same-player × component interactions reaches significance. Standard errors are two-way cluster-robust, clustered on the player taking the follow-up pass and match.

***Table S7.*** *Study 2 Model 4: Same-player effect with pass-difficulty covariates added.*

| **Predictor** | ***β*** | ***SE*** | ***OR*** | ***95% CI*** | ***p*** |
| --- | --- | --- | --- | --- | --- |
| Intercept | -3.344 | 0.127 | 0.035 | [0.028, 0.045] | < .001 *** |
| Position: AM/W (vs CM) | 0.287 | 0.053 | 1.332 | [1.202, 1.477] | < .001 *** |
| Position: CB (vs CM) | -0.414 | 0.055 | 0.661 | [0.593, 0.737] | < .001 *** |
| Position: DM (vs CM) | -0.208 | 0.071 | 0.812 | [0.707, 0.933] | .003 ** |
| Position: FB (vs CM) | -0.653 | 0.054 | 0.521 | [0.469, 0.578] | < .001 *** |
| Position: GK (vs CM) | -0.725 | 0.113 | 0.484 | [0.388, 0.604] | < .001 *** |
| Position: ST (vs CM) | 0.363 | 0.068 | 1.438 | [1.258, 1.643] | < .001 *** |
| Same player | 0.030 | 0.036 | 1.030 | [0.961, 1.105] | .402 |
| Pressure index | 0.042 | 0.022 | 1.042 | [0.998, 1.089] | .060 |
| Log pass length | 0.204 | 0.036 | 1.226 | [1.142, 1.317] | < .001 *** |
| Forward pass | 1.167 | 0.041 | 3.212 | [2.963, 3.482] | < .001 *** |

*Note.* As Table S4 with three pass-difficulty covariates added. The same-player coefficient is fully absorbed (p = .384) while the pass-difficulty effects carry large and highly significant coefficients. N = 54,409; no rows excluded for missing pass-difficulty fields in the pass-only sample. Standard errors are two-way cluster-robust, clustered on the player taking the follow-up pass and match.

**Study 3 Supplementary Materials**

***Table S8.*** *Observed unforced-pass error and success rates by chain depth.*

| **Depth** | **n (error)** | **% (err)** | **Error rate** | **Δ d0 (pp)** | **n (success)** | **% (suc)** | **Success rate** | **Δ d0 (pp)** |
| --- | --- | --- | --- | --- | --- | --- | --- | --- |
| 0 | 220,189 | 80.84 | 0.177 | +0.00 | 52,192 | 19.16 | 0.738 | +0.00 |
| 1 | 39,258 | 14.41 | 0.241 | +6.38 | 44,083 | 16.18 | 0.765 | +2.69 |
| 2 | 8,958 | 3.29 | 0.300 | +12.22 | 32,602 | 11.97 | 0.787 | +4.91 |
| 3 | 2,523 | 0.93 | 0.361 | +18.38 | 24,895 | 9.14 | 0.803 | +6.50 |
| 4 | 853 | 0.31 | 0.392 | +21.43 | 19,437 | 7.14 | 0.817 | +7.91 |
| 5 | 323 | 0.12 | 0.477 | +29.95 | 15,394 | 5.65 | 0.829 | +9.10 |
| 6 | 277 | 0.10 | 0.516 | +33.90 | 83,778 | 30.76 | 0.873 | +13.57 |

*Note.* Chain depth = number of consecutive immediately preceding unforced pass actions of the same outcome (error or success) by the same player in the same match. Depth 0 indicates the prior outcome was of the opposite type. Depth-6 row aggregates depth ≥ 6. Sample is the Study 3 primary dataset (N = 272,381 unforced pass actions; identical to the Study 1 primary sample).

***Table S8b.*** *Uncapped success-chain depth breakdown and severity thresholds.*

| **Depth** | **n** | **% of sample** |
| --- | --- | --- |
| 0 | 52,192 | 19.161 |
| 1 | 44,083 | 16.184 |
| 2 | 32,602 | 11.969 |
| 3 | 24,895 | 9.140 |
| 4 | 19,437 | 7.136 |
| 5 | 15,394 | 5.652 |
| 6 | 12,377 | 4.544 |
| 7 | 10,016 | 3.677 |
| 8 | 8,318 | 3.054 |
| 9 | 6,952 | 2.552 |
| 10 | 5,868 | 2.154 |
| 11 | 4,972 | 1.825 |
| 12 | 4,254 | 1.562 |
| 13 | 3,654 | 1.342 |
| 14 | 3,123 | 1.147 |
| 15 | 2,723 | 1.000 |
| 16+ | 21,521 | 7.901 |

**Severity thresholds (extending further into the tail):**

| **Depth threshold** | **n** | **% of sample** |
| --- | --- | --- |
| ≥ 6 | 83,778 | 30.758 |
| ≥ 7 | 71,401 | 26.214 |
| ≥ 10 | 46,115 | 16.930 |
| ≥ 15 | 24,244 | 8.901 |
| ≥ 20 | 13,668 | 5.018 |
| ≥ 25 | 8,174 | 3.001 |

*Note.* Raw (uncapped) success_chain_depth distribution, extending Table S8's depth-6-capped bin. The depth-6-and-above bin used for modelling (Tables S8-S10) aggregates 30.76% of the sample on the success side, versus 0.10% on the error side at the same cap -- the two capped variables are not comparably restrictive. The tail remains substantial well beyond depth 6 (16.93% of the sample at depth ≥ 10; 3.00% at depth ≥ 25). Maximum observed raw success_chain_depth = 95. See Table S8c for a severity-matched sensitivity refit and Table S8d for a model-free check of whether the underlying relationship is linear.

***Table S8c.*** *Success-chain sensitivity analysis: severity-matched capping refit.*

| **Specification** | **Cap** | **Severity (% ≥ cap)** | **OR/step** | **Player slope SD** | **Corr(int, slope)** |
| --- | --- | --- | --- | --- | --- |
| Success — primary (reported) | 6 | 30.8% | 1.030 | 0.044 | −0.203 |
| Success — severity-matched | 65 | 0.09% | 1.017 | 0.012 | −0.495 |
| Error — for reference | 6 | 0.10% | 1.066 | 0.093 | −0.599 |

*Note.* Severity-matched cap (65) is the success-chain depth at which capping severity most closely approximates the error side's severity at its cap of 6 (see Table S8b). Under this fairer comparison, the success-side OR/step falls from 1.030 to 1.017 and the player random-slope SD falls from 0.044 to 0.012 (a ~4-fold reduction), while the intercept-slope correlation strengthens from −.203 to −.495 — closer to, though still smaller in magnitude than, the error side's −.599. This indicates the primary (cap-6) estimate overstates success-side between-player heterogeneity in chain susceptibility: the true asymmetry between error- and success-chain individual differences is larger than the primary analysis alone suggests. See Table S8d for a model-free check of whether the underlying relationship is linear at all.

***Table S8d.*** *Observed success rate by chain-depth bin and log-odds scale, testing the linearity assumption.*

| **Bin** | **Depth midpoint** | **n** | **Success rate** | **Logit** | **Δ rate / unit depth** | **Δ logit / unit depth** |
| --- | --- | --- | --- | --- | --- | --- |
| 0 | 0 | 52,192 | 0.7377 | 1.0341 | — | — |
| 1 | 1 | 44,083 | 0.7646 | 1.1783 | +0.02693 | +0.14419 |
| 2 | 2 | 32,602 | 0.7869 | 1.3061 | +0.02221 | +0.12773 |
| 3 | 3 | 24,895 | 0.8027 | 1.4035 | +0.01588 | +0.09739 |
| 4 | 4 | 19,437 | 0.8168 | 1.4951 | +0.01411 | +0.09166 |
| 5 | 5 | 15,394 | 0.8288 | 1.5769 | +0.01192 | +0.08178 |
| 6 | 6 | 12,377 | 0.8311 | 1.5931 | +0.00229 | +0.01625 |
| 7-15 | 11 | 49,880 | 0.8676 | 1.8796 | +0.00730 | +0.05729 |
| 16-30 | 23 | 16,895 | 0.9046 | 2.2493 | +0.00309 | +0.03081 |
| 31-65 | 48 | 4,410 | 0.9363 | 2.6874 | +0.00127 | +0.01753 |
| 66+ | 80 | 216 | 0.9306 | 2.5953 | -0.00018 | -0.00288 |

*Note.* Observed (model-free) success rate and its log-odds (logit) transform within each success-chain-depth bin, with the per-unit-of-depth change from the preceding bin. If the relationship were linear in log-odds, the Δ logit / unit depth column would be roughly constant across rows. Instead it falls steadily from +0.144 (depth 0→1) to +0.018 (depth 23→48), consistent with a saturating relationship that plateaus by roughly depth 30–50, rather than continuing to escalate at the rate observed over depths 0–6. This is a model-free complement to Table S8c and does not depend on any capping or contrast-coding choice.

***Table S9.*** *GLMM fixed-effect chain slopes for error and success chains (Models 1 and 2).*

| **Side** | **Model** | **Intercept** | **Chain slope** | ***SE*** | ***p*** | **OR/step** | **N** |
| --- | --- | --- | --- | --- | --- | --- | --- |
| Error | Model 1 | -1.217 | 0.064 | 0.008 | < .001 | 1.066 | 272,381 |
| Error | Model 2 | -2.110 | 0.017 | 0.009 | .065 | 1.017 | 272,381 |
| Success | Model 1 | 1.128 | 0.029 | 0.003 | < .001 | 1.030 | 272,381 |
| Success | Model 2 | 2.058 | 0.021 | 0.003 | < .001 | 1.022 | 272,381 |

*Note.* Binomial generalised linear mixed models fitted in R with lme4 (bobyqa optimiser, Laplace approximation). Model 1 includes chain depth, position fixed effects, a random intercept and random slope on chain depth at the player level, and a random intercept at the match level. Model 2 adds three pass-difficulty covariates (log pass length, forward-pass indicator, pass-height category). Intercept and chain slope are reported on the log-odds scale; OR/step is the multiplicative odds change per consecutive-prior-outcome step.

***Table S10.*** *GLMM variance components and random-effect structure for error and success chains.*

| **Side** | **Model** | **Player int. SD** | **Player slope SD** | **Corr(int, slope)** | **Match int. SD** | **LRT χ²** | ***p*** |
| --- | --- | --- | --- | --- | --- | --- | --- |
| Error | Model 1 | 0.480 | 0.093 | −0.599 | 0.259 | 52.3 | < .001 |
| Error | Model 2 | 0.357 | 0.095 | −0.315 | 0.164 | — | — |
| Success | Model 1 | 0.450 | 0.044 | −0.203 | 0.253 | 58.6 | < .001 |
| Success | Model 2 | 0.352 | 0.042 | −0.353 | 0.159 | — | — |

*Note.* Random-effects standard deviations and correlations from the Study 3 GLMMs. LRT statistic compares the random-intercept-only model to the random-intercept-plus-random-slope model on chain depth (Model 1). The substantial player random-slope SD on the error side and its strong negative correlation with the random intercept constitute the model-implied evidence for the baseline error tendency – chain susceptibility relationship reported in the Results.

***Table S11.*** *Spearman rank correlation between intercept and slope BLUPs across a monotonic observation-threshold sweep, error and success sides.*

| **Min obs (depth ≥ 1)** | **n (error)** | **r (error)** | **p (error)** | **n (success)** | **r (success)** | **p (success)** |
| --- | --- | --- | --- | --- | --- | --- |
| ≥ 5 | 1,685 | −0.920 | < .001 | 1,685 | −0.117 | < .001 |
| ≥ 10 | 1,289 | −0.908 | < .001 | 1,289 | −0.124 | < .001 |
| ≥ 15 | 998 | −0.893 | < .001 | 998 | −0.134 | < .001 |
| ≥ 20 | 750 | −0.878 | < .001 | 750 | −0.144 | < .001 |
| ≥ 30 | 465 | −0.854 | < .001 | 465 | −0.159 | < .001 |
| ≥ 50 | 191 | −0.812 | < .001 | 191 | −0.130 | .072 |
| ≥ 75 | 96 | −0.787 | < .001 | 96 | −0.160 | .120 |
| ≥ 100 | 59 | −0.808 | < .001 | 59 | −0.095 | .472 |

*Note.* Spearman rank correlation between empirical-Bayes BLUPs for the player random intercept and random slope on chain depth, extracted from Study 3 Model 1, computed identically for both error and success sides across a single monotonic sweep of the minimum-observations-at-chain-depth-≥-1 inclusion criterion. The threshold used for the primary single-sample analyses elsewhere (Figure 6, Table S12) is ≥ 10, unchanged. The error-side correlation is strong and stable across the entire range tested; the success-side correlation is an order of magnitude smaller throughout and loses statistical significance once the sample becomes sparse (≥ 50 and above) — a stability contrast that is itself informative, not just a magnitude difference. Note the empirical BLUP correlation is shrinkage-driven; the model-implied correlation from the random-effects covariance matrix is reported in Table S10.

***Figure S2.*** *Stability of the intercept-slope BLUP correlation across inclusion thresholds, error vs. success chains.*

*Note.* Spearman r (Table S11) plotted against the minimum-observations inclusion threshold, for both sides. The error-chain correlation (red) is essentially flat and strongly negative across the entire range; the success-chain correlation (blue) stays close to zero throughout and is not reliably distinguishable from the flat line at the sparser thresholds.

***Table S12.*** *Within-position Spearman correlations between intercept and slope BLUPs (≥ 10 observations at chain depth ≥ 1, error side).*

| **Position group** | **n players** | **Spearman r (intercept BLUP × slope BLUP)** |
| --- | --- | --- |
| Overall | 1,289 | −0.908 |
| Goalkeeper | 134 | −0.867 |
| Centre-back | 221 | −0.953 |
| Full-back | 306 | −0.880 |
| Defensive midfielder | 94 | −0.906 |
| Central midfielder | 278 | −0.934 |
| Attacking midfielder / Winger | 131 | −0.909 |
| Striker | 125 | −0.855 |

*Note.* Spearman rank correlations within each of the seven position groups in the primary sample (players with ≥ 10 observations at chain depth ≥ 1). Each within-position correlation is strongly negative, indicating that the baseline error tendency – chain susceptibility relationship holds within position categories rather than being driven by between-position composition.

***Table S13.*** *Behavioural signatures within error chains, by intercept BLUP tertile.*

| **Tertile** | **Depth** | **n** | **Mean log pass len** | **Forward rate (%)** | **High rate (%)** |
| --- | --- | --- | --- | --- | --- |
| Low-error (low intercept) | 0 | 140,548 | 2.889 | 63.6 | 13.8 |
|  | 1 | 18,131 | 2.898 | 63.8 | 19.4 |
|  | 2 | 2,980 | 2.965 | 67.0 | 26.1 |
|  | 3 | 603 | 3.097 | 70.6 | 32.3 |
|  | 4 | 150 | 3.258 | 81.3 | 45.3 |
|  | 5 | 57 | 3.435 | 86.0 | 52.6 |
|  | 6 | 33 | 3.601 | 93.9 | 66.7 |
| Mid-error | 0 | 38,741 | 2.942 | 64.7 | 25.5 |
|  | 1 | 9,870 | 2.950 | 65.5 | 29.6 |
|  | 2 | 2,558 | 3.014 | 68.4 | 31.3 |
|  | 3 | 739 | 3.137 | 74.0 | 41.5 |
|  | 4 | 265 | 3.230 | 80.8 | 45.3 |
|  | 5 | 83 | 3.445 | 80.7 | 65.1 |
|  | 6 | 62 | 3.729 | 93.5 | 77.4 |
| High-error (high intercept) | 0 | 23,093 | 3.012 | 69.5 | 32.7 |
|  | 1 | 8,180 | 3.016 | 69.1 | 36.1 |
|  | 2 | 2,825 | 3.081 | 71.5 | 39.8 |
|  | 3 | 1,040 | 3.199 | 74.2 | 47.8 |
|  | 4 | 399 | 3.165 | 77.7 | 47.1 |
|  | 5 | 169 | 3.467 | 85.8 | 65.7 |
|  | 6 | 179 | 3.578 | 89.9 | 59.2 |

*Note.* Behavioural signatures within error chains by player baseline error tendency tertile. Players are stratified by Model 1 (error) random intercept BLUP into tertiles (low-error → high-error). Within each tertile, the mean log pass length, the percentage of passes that are forward, and the percentage that are aerial (high) are reported across error-chain depths. Sample is the Study 3 primary sample (players with ≥ 10 observations at chain depth ≥ 1; n = 1,289 players).

***Table S14.*** *Formal tests of behavioural-signature slope differences across baseline error tendency tertiles within error chains.*

| **Outcome** | **Estimator** | **Mid-error slope** | **Low-error slope** | **High-error slope** | **Low-error vs Mid-error p** | **High-error vs Mid-error p** |
| --- | --- | --- | --- | --- | --- | --- |
| Log pass length | OLS | 0.0521 | 0.0392 | 0.0545 | 0.533 | 0.905 |
| Forward-pass rate | Logistic | 1.12 | 1.073 | 1.095 | 0.291 | 0.533 |
| High (aerial) pass rate | Logistic | 1.246 | 1.486 | 1.202 | < .001*** | 0.347 |

*Note.* Formal tests of whether the behavioural-signature slopes across error chain depth differ between baseline error tendency tertiles within the Study 3 primary sample. For log pass length the estimator is ordinary least squares (slopes are in log-units per chain-depth step); for forward-pass and high-pass rate the estimator is logistic regression (slopes are reported as odds ratios per chain-depth step). All three models use two-way cluster-robust standard errors, clustered on player and match. Mid-error-tertile players serve as the reference category; the right-most two columns test whether the low-error and high-error tertile slopes differ from the mid-error-tertile slope. *** p < .001.

***Table S15.*** *Baseline error tendency and chain susceptibility: random-effects parameters before and after pass-difficulty adjustment (error side).*

| **Model** | **Intercept SD** | **Slope SD** | **Model-implied r** | **BLUP Spearman r** | ***p*** | **n** |
| --- | --- | --- | --- | --- | --- | --- |
| Model 1 (unadjusted) | 0.480 | 0.093 | -0.599 | -0.908 | < .001 | 1,289 |
| Model 2 (+ pass difficulty) | 0.357 | 0.095 | -0.315 | -0.578 | < .001 | 1,289 |

*Note.* Random-effects standard deviations and intercept-slope correlations for the error-side models, before and after adding the three pass-difficulty covariates. Model-implied r derives from the estimated random-effects covariance; BLUP Spearman r is the rank correlation between fitted player intercept and slope BLUPs. Pass-difficulty adjustment reduces the intercept standard deviation by 25.6% (a 44.7% reduction in intercept variance, as reported in the main text) and the intercept-slope covariance by 60.3%, while the slope standard deviation is essentially unchanged. Generated by step4c_m2_correlation.R.
